# Single nucleotide resolution RNA-seq uncovers new regulatory mechanisms in the opportunistic pathogen *Streptococcus agalactiae*

**DOI:** 10.1186/s12864-015-1583-4

**Published:** 2015-05-30

**Authors:** Isabelle Rosinski-Chupin, Elisabeth Sauvage, Odile Sismeiro, Adrien Villain, Violette Da Cunha, Marie-Elise Caliot, Marie-Agnès Dillies, Patrick Trieu-Cuot, Philippe Bouloc, Marie-Frédérique Lartigue, Philippe Glaser

**Affiliations:** Institut Pasteur, Unité de Biologie des Bactéries Pathogènes à Gram Positif, 28 rue du Docteur Roux, 75724, Paris Cedex 15, France; CNRS UMR 3525, Paris, France; Institut Pasteur, Transcriptome and Epigenome Platform, 28 rue du Docteur Roux, 75724, Paris Cedex 15, France; Institute for Integrative Biology of the Cell (I2BC), CEA, CNRS, Université Paris-Sud, bâtiment 400, 91405 Orsay, France; Université de Tours, UMR1282 Infectiologie et Santé Publique, F-37000 Tours, France; CHRU de Tours, F-37044 Tours, France; INRA, UMR1282 Infectiologie et Santé Publique, F-37380 Nouzilly, France

**Keywords:** Deep sequencing, Reiterative transcription, Riboswitches, Non coding RNAs, csRNAs, Promoters, CRISPR, Antisense transcription, Operon

## Abstract

**Background:**

*Streptococcus agalactiae*, or Group B *Streptococcus*, is a leading cause of neonatal infections and an increasing cause of infections in adults with underlying diseases. In an effort to reconstruct the transcriptional networks involved in *S. agalactiae* physiology and pathogenesis, we performed an extensive and robust characterization of its transcriptome through a combination of differential RNA-sequencing in eight different growth conditions or genetic backgrounds and strand-specific RNA-sequencing.

**Results:**

Our study identified 1,210 transcription start sites (TSSs) and 655 transcript ends as well as 39 riboswitches and cis-regulatory regions, 39 cis-antisense non-coding RNAs and 47 small RNAs potentially acting in trans. Among these putative regulatory RNAs, ten were differentially expressed in response to an acid stress and two riboswitches sensed directly or indirectly the pH modification. Strikingly, 15% of the TSSs identified were associated with the incorporation of pseudo-templated nucleotides, showing that reiterative transcription is a pervasive process in *S. agalactiae.* In particular, 40% of the TSSs upstream genes involved in nucleotide metabolism show reiterative transcription potentially regulating gene expression, as exemplified for *pyrG* and *thyA* encoding the CTP synthase and the thymidylate synthase respectively.

**Conclusions:**

This comprehensive map of the transcriptome at the single nucleotide resolution led to the discovery of new regulatory mechanisms in *S. agalactiae*. It also provides the basis for in depth analyses of transcriptional networks in *S. agalactiae* and of the regulatory role of reiterative transcription following variations of intra-cellular nucleotide pools.

**Electronic supplementary material:**

The online version of this article (doi:10.1186/s12864-015-1583-4) contains supplementary material, which is available to authorized users.

## Background

*Streptococcus agalactiae*, or Group B *Streptococcus*, is a Gram-positive bacterium known as a commensal of the digestive and genitourinary tracts of 10–30% of the human population [1]. It emerged during the 1960s as a leading cause of neonatal infections, causing pneumonia, septicemia and meningitis [2,3]. It also represents an increasing cause of infections in the elderly and in adults with underlying diseases [4]. In addition, *S. agalactiae* is frequently associated with animal diseases, being responsible for bovine mastitis [5] and for massive epidemic outbreaks in fish farms [6]. While a number of *S. agalactiae* factors involved in host colonization or in virulence have been described [7,8], the transcriptional networks coordinating their expression during the progression from commensalism to virulence, or in response to changing host environments, are still largely unknown [9,10]. As many as 93 putative transcriptional regulators and 17–20 two-component regulatory systems (TCSs) are annotated in *S. agalactiae* genomes [11,12]. Five TCSs (CovR/CovS, DltR/DltS, RgfC/RgfA, FspR/FspS and CiaR/CiaH) were shown to control the expression of virulence factors [13-17]. In particular, the CovRS system, also called CsrRS, is a major regulator of gene expression in *S. agalactiae*, governing directly or indirectly the expression of more than 7% of the genome, including many virulence associated genes [14]. The expression of the CovR/CovS-controlled genes was found to be deeply modified in response to environmental variations such as shifts to acidic pH [18]. *S. agalactiae* encounters acidic pH at crucial steps of the colonization and invasion processes, such as during the adaptation to the vaginal cavity or during intracellular survival in macrophages [19].

Over the past ten years, our understanding of bacterial transcription has greatly advanced thanks to the combination of high-throughput technologies, namely tiling arrays and deep RNA sequencing (RNA-seq) with classical genetics and biochemical assays. In particular, RNA-seq has offered tremendous power for high-resolution transcriptome characterization, allowing both differential-expression analysis and identification of new transcripts not predicted by bioinformatics. Characterization of the primary transcriptome, which is a catalog of all expressed RNA molecules that carry a 5′-triphosphate group indicative of transcription start sites (TSSs), has also greatly facilitated the identification of promoter regions and their interspecies comparisons [20]. These genome-wide transcriptome studies revealed a widespread antisense transcription, frequent read-through of terminators and transcription initiations inside cistrons, which altogether challenged the conventional view of the operon [20-23]. They also identified dozens of novel small RNAs (sRNAs) potentially involved in the regulation of gene expression by interfering with RNA transcription, translation and stability [24,25]. As their target genes were characterized, these sRNAs emerged as key regulators of metabolic, physiological and pathogenic processes.

In this work, we aimed at characterizing the transcriptional landscape of *S. agalactiae* by a thorough identification of the promoter regions and operon structure, as well as of sRNAs and cis regulatory 5′ untranslated regions (5′UTR). We combined differential RNA-seq (dRNA-seq), and strand-specific RNA-seq on multiple RNA samples from the model strain NEM316 to maximize the identification of 5′ and 3′ ends. This revealed an unexpected high number of TSSs associated with a reiterative transcription suggesting new potential regulatory mechanisms. Among 39 cis-regulatory regions identified, eleven were novel and two were found to respond to acidic conditions. Ten out of 47 intergenic sRNAs were also predicted to be involved in *S. agalactiae* response to acid stress. This comprehensive characterization of *S. agalactiae* transcriptional landscape paves the way for further deciphering the regulatory networks that coordinate gene expression during the progression from commensalism to virulence.

## Results and discussion

### Genome-wide mapping of TSSs maximized by multiple differential RNA-seq

To globally identify TSSs in *S. agalactiae*, we used a differential RNA-seq (dRNA-seq) strategy, based on selective Tobacco Acid Pyrophosphatase (TAP) treatment and 5′ adapter ligation, which differentiates primary transcripts and processed RNAs [26,27]. To increase the sensitivity and the robustness of TSS detection, we performed four independent experiments with RNA extracted from strain NEM316 grown under different growth or stress conditions (Additional file 1): mid-exponential and late-exponential growth phases in a rich culture medium (TH) and acid stress condition. The last sample was a mix of RNAs prepared at mid-exponential and stationary growth phases in rich culture medium and at the beginning of stationary phase in a poor culture medium. In addition, we performed TSS mapping on three NEM316 derivatives in which the *ciaRH*, *relRS* or *covRS* TCS loci were deleted. As these TCS may act as positive or negative regulators depending on their targets, we speculated that their inactivation would increase the transcription of genes weakly or not expressed in the WT strain under the conditions tested. Two growth conditions (early and late exponential phases) were used for the *covRS* mutant, while the *relRS* and *ciaRH* mutants were grown to early or late exponential phases, respectively.

**Figure 1:**
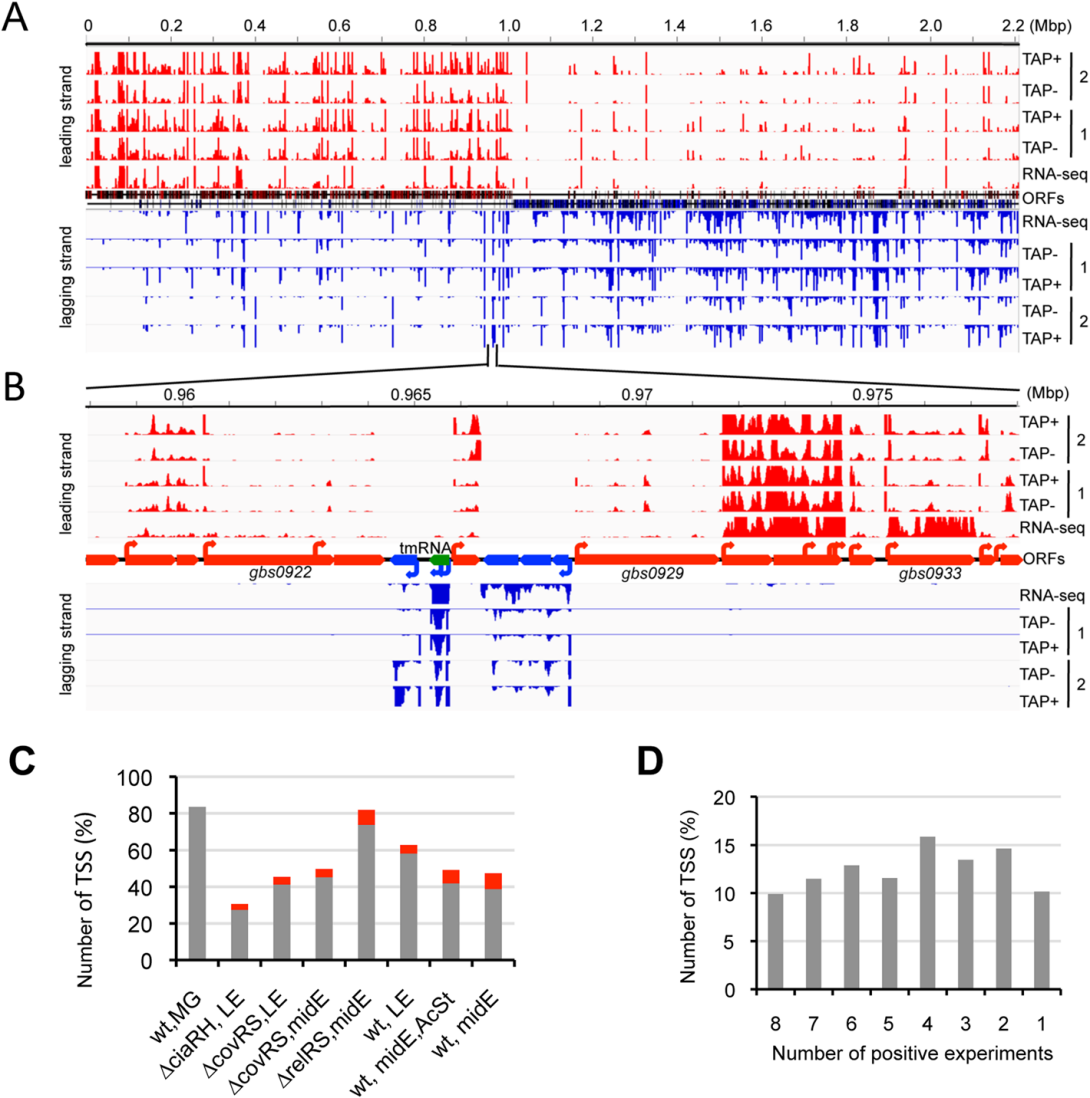
Characterization of transcription start sites in *S. agalactiae.* **A**. Visualization of sequence reads mapped to the genome of strain NEM316 in conditions of dRNA-seq: strand-specific sequencing of transcript 5′ ends with (TAP+) and without (TAP-) TAP treatment, and strand-specific RNA-seq. Two dRNA-seq experiments are shown corresponding to 1: RNA from multiple growth conditions (MG sample); 2: RNA from a *∆covRS* mutant grown to mid-exponential phase. The RNA-seq library was prepared with the wt strain at mid-exponential phase. **B**. Detailed view of the 958000–978000 region. Protein coding genes annotated on the (+) and (−) strands are indicated by red and blue large arrows. TSSs are depicted as small arrows. Based on dRNA-seq and RNA-seq data, a transcript corresponding to a ncRNA (srn040/tmRNA) was annotated and is shown as a large green arrow*.* **C**. Proportions of the total TSSs detected under each experimental condition. Grey: TSSs detected with RNA from multiple growth conditions (wt, MG); red: TSSs not detected in wt, MG. MG: mixture of growth conditions; LE: late exponential phase; midE: mid-exponential growth phase; AcStr: acid stress. **D**: Proportions of TSSs according to the number of experiments in which they were detected.

A total of ~220 million sequence reads were generated under TAP+ and TAP- conditions for the 16 libraries (Additional file 1). Reads were aligned on the *S. agalactiae* NEM316 genome sequence [11], with 4.7 and 2.3 million reads on average aligning to non-ribosomal regions under TAP+ and TAP- conditions, respectively. The density of reads aligned on the chromosome was higher on the leading strand (Figure 1A). This reflects the general bias in gene orientation in *S. agalactiae*, with 81% of the coding sequences transcribed in the same direction as the movement of the replication fork. TAP+ and TAP- profiles were similar except at discrete positions, mostly found close to the annotated start codon of coding sequences (CDSs), where the number of reads under TAP+ conditions largely exceeded that under TAP- conditions (Figure 1B). This difference was used to discriminate between native and processed RNA ends and to predict TSSs. The large number of sites corresponding to processed ends was used to estimate the dispersion parameters of the experiments and to infer a statistical significance to the TAP+/TAP- differences at each position, as described in the “Methods” section. Combination of the TSSs identified under the eight experimental conditions led to a final list of 1,106 TSSs determined with high confidence. In addition 104 TSSs predicted with a lower degree of confidence from the dRNA-seq experiments were confirmed by the RNA-seq analysis (see below). 80% of the 1,210 TSSs were detected in the RNA sample pool corresponding to the mixture of growth conditions (Figure 1C) and 60% of the TSSs were predicted under at least four conditions (Figure 1D). Globally, the sensitivity of detection was strongly improved by combining growth conditions and by including mutants of transcriptional regulators. The position of the 1,210 TSSs and the normalized number of TAP+ reads associated to these TSSs under the eight conditions are given in Additional file 2.

TSSs were further classified according to their position relative to the CDSs. 75% (n = 927) were TSSs upstream protein-coding genes, among which 891 corresponded to primary TSS and 36 to secondary TSS. We identified 34 and two protein-coding genes preceded by two promoters and three promoters, respectively. The start codons for 11 genes, in which the TSS was downstream the previously predicted translation initiation codon, were corrected and two new potentially coding genes were annotated. 225 TSSs were located inside CDS, 191 initiating transcription in the same orientation as the CDS and 34 in the opposite orientation. Five supplementary TSS, close to CDS (<100 nt) but in opposite orientation also initiated an antisense transcription. Finally 53 TSSs in intergenic regions were upstream potential sRNA and tRNA genes (Table 1).

**Table 1:** **Main characteristics of the** ***S. agalactiae*** **transcriptome (strain NEM316)**

**Genetic features**	**Number**	**Comments**
***Genes and pseudogenes***	***2207***	
Coding genes	2084	2082 previously annotated CDS, including 11 for which the initiation codon has been reannotated; 2 new CDS identified in this study
Pseudogenes	36	
rRNA operons	7	
tRNAs genes	80	14 located outside the rRNA regions
***TSS identified in the study***	**1210**	
Primary TSS	891	
Secondary TSS	36	
TSS internal to CDS, same orientation as CDS	191	
TSS initiating antisense transcription	39	34 TSS inside CDS, 5 TSS located close to CDS
Intergenic TSS	53	including 8 TSS upstream tRNAs genes and one upstream a rRNA operon
***Sigma70 promoters***	***1179***	
***Transcript ends***	***655***	602 corresponding to predicted rho-independent terminators
***Transcription units***	***891***	
Operons identified in the study	407	including 56 operons with internal promoters, 26 operons with internal terminators and 15 operons with both internal promoters and internal terminators
Monocistrons	484	
***sRNAs identified in the study***	**120**	
Cis-regulatory sequences	39	23 belonging to rfam families and acting through attenuation of transcription
		5 belonging to rfam families and acting through regulation of translation (no sRNA)
		11 not belonging to rfam families and acting through attenuation of transcription
Cis-antisense sRNAs	39	
Trans acting sRNAs	47	44 with TSS in intergenic regions, 1 with TSS at the end of a transcriptionally inactive gene and 2 likely resulting from the cleavage of longer transcripts

### Canonical sigma70 promoters are major determinants of transcription initiation in *S. agalactiae*

**Figure 2:**
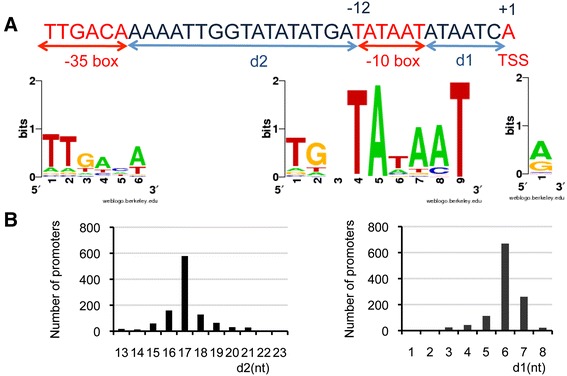
The vast majority of *S. agalactiae* promoters are sigma70 dependent. **A**. Motif search upstream of 1179/1210 *S. agalactiae* TSSs reveals extended Pribnow or −10 boxes and less conserved −35 boxes. The consensus sequence was generated using WebLogo (http://weblogo.berkeley.edu/logo.cgi). **B**. Mean distances between the −10 box and the TSS (d1) and between the −10 and −35 boxes (d2). Distances were calculated between the 3′ end nucleotide of the −10 box and the TSS and between the 3′ nucleotide of the −35 box and the 5′ nucleotide of the TATAAT sequence.

By analyzing the 50 nucleotides upstream each TSS, we predicted promoter sequences related to the prototype Sigma70 promoter (TTGACA-X_15/21bp_-TATAAT) [28]) upstream 90% of the TSSs (1091 promoters) (Table 1, Additional file 2 and Figure 2). For 88 (7%) additional promoters, only the consensus Sigma70 -10 box was identified. 64% of all the promoters had a TGn sequence extending the −10 consensus sequence. This high frequency of Sigma70 promoters is in agreement with the identification of only three sigma factors in the genome sequence of strain NEM316: the housekeeping Sigma70, ComX and an ECF-type sigma factor [11]. For approximately 70% of the promoters, transcription initiation occurred at a single nucleotide located on average six nucleotides downstream the TATA box (Figure 2B). The initiating nucleotide was A (63%) or G (32%) reflecting the preference of the RNA polymerase for purine residues as initiator nucleotides, as observed in *Bacillus subtilis* and *Escherichia coli* [28,29]. For the remaining 30% of the promoters, initiation arose at two to six tightly clustered nucleotides resulting from an alternative choice of the initiating nucleotide for half of the cases or from the incorporation of pseudo-templated nucleotides, which modified the apparent TSS position.

### Reiterative initiation of transcription is frequent in *S. agalactiae*

Reiterative transcription, also known as transcript slippage or pseudo-templated transcription was documented as an intrinsic property of RNA polymerase from as early as 1964 [30]. However, as it has been studied on a gene-by-gene basis, only a few examples have been reported to date in bacteria. Its functional importance as a regulatory mechanism was demonstrated in the transcription of several genes involved in nucleotide metabolism such as *pyrB1*, *pyrG, carAB*, *codBA*, *upp-uraA* in *E. coli* or *B. subtilis* and for the *gal* operon in *E. coli* [31-33]*.* We developed a method to mine dRNA-seq data for reiterative transcription at a genome-wide level. Indeed, while the RNA-polymerase stuttering at a promoter may lead to non-productive transcription, the switch to a non-reiterative nucleotide addition will generate a transcript differing in its 5′ end from its DNA-template, by one or several nucleotides detectable by RNA-seq.

**Figure 3:**
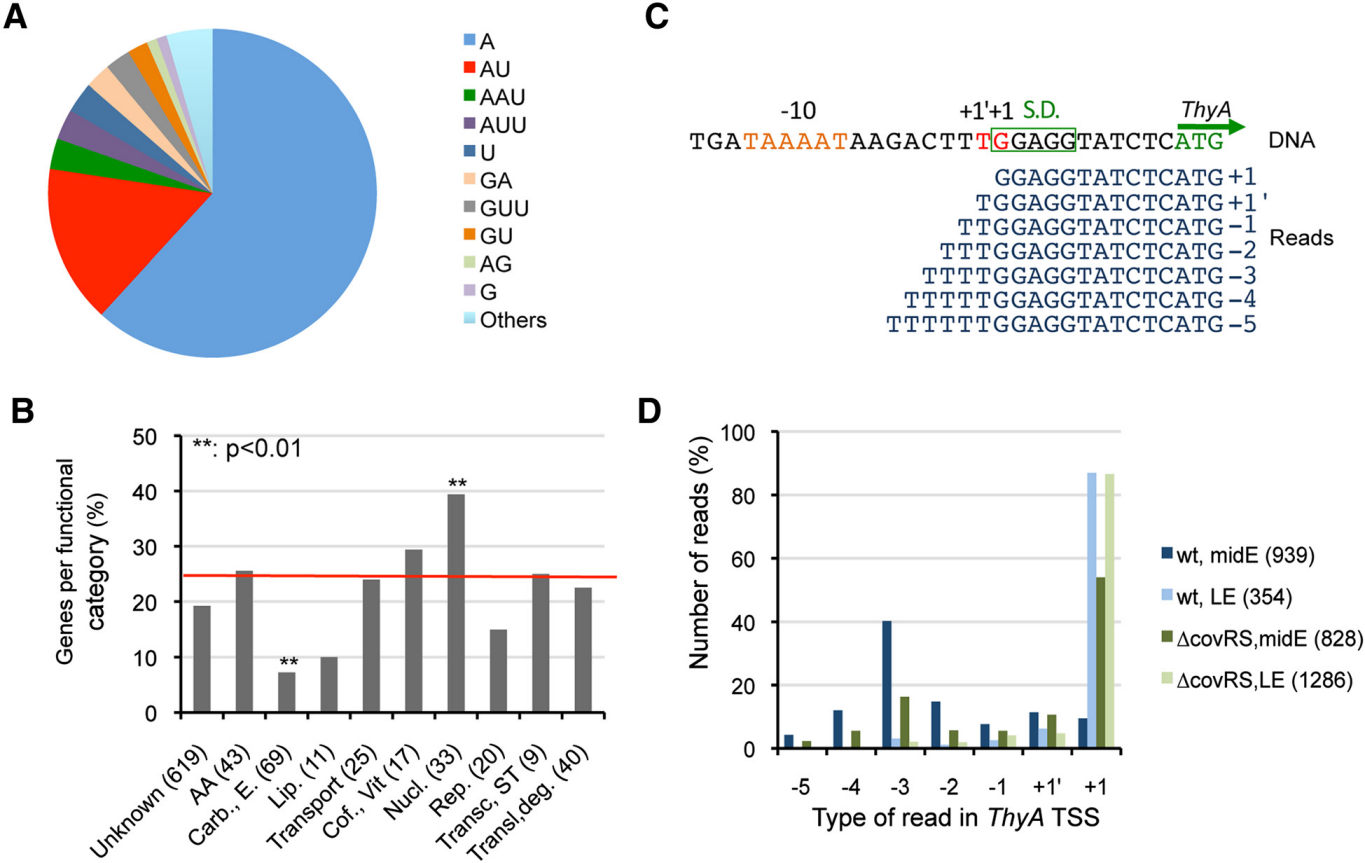
Extensive reiterative transcription in *S. agalactiae.* **A**. Pie chart of the occurrence of pseudo-templated nucleotides at the transcription initiation sites. **B**. Functional classification of genes submitted to reiterative transcription according to the KEGG categories (AA: Amino acid metabolism; Carb.,E.: carbohydrate, glycan and energy metabolism; Lip.: Lipid metabolism; Transport: Membrane transport; Cof.,Vit: metabolism of cofactors and vitamins; Nucl.: Nucleotide metabolism; Rep.: Replication and Repair; Transc, ST: Transcription, Signal transduction; Transl., deg.: translation, protein folding, sorting and degradation; Unknown: genes for which the KEGG category was not defined). TSSs were classified according to the functional class of the first gene of the transcription unit. The number of TSSs linked to each class is indicated in brackets. The mean proportion of TSSs with reiterative transcription is indicated by the red line. The proportion of genes showing reiterative transcription was found to be significantly higher (p < 0.01) for genes involved in nucleotide metabolism and lower (p < 0.01) for genes involved in carbohydrate, glycan and energy metabolism using chi-square and Fisher exact tests. **C**. Reiterative transcription at the *thyA* gene. The promoter of *thyA* directs initiation of transcription at a T or a G (shown in red on the non-template strand of the DNA sequence). The G position also corresponds to the first nucleotide of the Shine-Dalgarno (SD) sequence. Initiation at the T position leads to reiterative incorporation of pseudo-templated U. **D**. The proportion of reads containing pseudo-templated nucleotides in *thyA* transcripts is found to vary depending on growth conditions: mid-exponential and late exponential phases in the wt and the ∆*covRS* mutant of strain NEM316. The total number of reads corresponding to the TSS is indicated in brackets next to the experimental condition in the figure legend.

By modifying our alignment protocol to systematically detect pseudo-templated nucleotides at transcript 5′ ends, we observed that up to 15% of *S. agalactiae* TSSs were associated with a reiterative transcription (Additional file 2). The proportion of transcripts with pseudo-templated nucleotides (5-100%) and the number of added nucleotides (up to 10) were variable among TSSs. These non-templated nucleotides, most often A repeats (67%) (Figure 3A) were generally associated with the presence of nucleotide stretches on the DNA template, as reported [34]. However, we also observed reiterative process involving di or tri-nucleotides, as noted in eukaryotes [35], with 15% of AU reiteration.

Analysis of genes subjected to reiterative initiation of transcription according to KEGG functional annotation revealed an enrichment in genes involved in nucleotide metabolism (Figure 3B), e.g., *pyrG* encoding the CTP synthetase. In *B. subtilis*, reiterative incorporation of pseudo-templated G nucleotides at *pyrG* TSS was shown to occur when cells are starved for pyrimidines and to prevent transcription attenuation [36]. In *S. agalactiae*, both a similar TSS sequence and a rho-independent terminator are detected upstream *pyrG* (Additional file 3), suggesting a similar regulatory mechanism. Another key enzyme in nucleotide biosynthesis, thymidylate synthetase (*thyA*), might also be regulated by reiterative transcription in *S. agalactiae*. Indeed, transcription of *thyA* occurs at a G or at the adjacent upstream T, where it leads to reiterative addition of up to six U residues (Figure 3C). The *thyA* gene has a short 12-nt long 5′UTR and the TSS corresponds to the first G residue of the Shine-Dalgarno sequence (GGAGG). This suggests a mechanism where, depending on the intracellular pools of GTP and UTP, the amplitude of reiterative transcription might modulate *thyA* mRNA translation initiation. Interestingly the proportion of RNAs with pseudo-templated nucleotides varied according to the growth condition tested, supporting that reiterative transcription has a regulatory function (Figure 3D).

Our results show for the first time that reiterative transcription is an extensive process in *S. agalactiae*. We only considered the incorporation of pseudo-templated nucleotides linked to the switch to productive transcription. Therefore our data provide a minimal estimate of the whole tendency of the polymerase at a given TSS sequence to enter into the reiterative mode and the true incidence of reiterative transcription is likely underestimated. Expression of several genes regulated by reiterative transcription-dependent mechanisms was found to be strongly affected during cellular stress created by intermediary metabolites imbalances in *E. coli* leading to variations in intracellular nucleotide pools [37]. Our results show that dRNA-seq may be used to capture some of the modifications at the transcript 5′ end, allowing a more systematical characterization of the metabolic factors that regulate gene expression by acting on the extent of repetitive nucleotide addition or on the choice of the initiating nucleotide.

### The *S. agalactiae* transcriptional landscape

2,082 protein coding genes, 36 pseudogenes, seven rRNA clusters and 80 tRNA genes were annotated in strain NEM316 genome [11]. To describe the operon organization, we combined to the TSS analysis whole transcript sequencing (RNA-seq). We chose a two-step adaptor ligation-based directional RNA-seq protocol, which provides transcript coverage in a strand-specific manner and permits a precise mapping of transcript 3′ ends [21]. RNA-seq analysis was performed on three independent biological replicates of exponentially growing bacteria submitted or not to a 20 minutes acid stress (pH 5.2). For each library more than ~6 million reads mapping to non-ribosomal regions were analyzed (Additional file 1). The number of reads mapped to each coding sequence (CDS) was corrected for gene length and library depth to generate normalized reads per kilobase (kb) per million mapped reads (RPKM). Under the conditions tested, 79% (1,682) of the annotated genes or pseudogenes of *S. agalactiae* were expressed, using a detection threshold of three RPKM. By analyzing the distribution of the reads along the genome sequence, we characterized the 3′ end of 655 transcripts (Table 1, Additional file 4). 92% (n = 602) of them correspond to rho-independent terminators predicted in the *S. agalactiae* genome [38] confirming that they are *bona fide* transcription termination sites.

Taking into account the information from TSS and transcript mapping, we predicted 407 polycistronic transcription units containing two to 23 genes (Table 1, Additional file 5). Seventy-one contain internal TSSs and 40 internal terminators and might be further divided into sub-operons (Table 1). This occurrence of internal promoters and terminators might lead to differential expression of the genes of the same operon as already noted in other genome-wide studies [20]. 75 additional internal TSSs (Additional file 2) were located in monocistrons or in the last gene of an operon. They might correspond to promoters of small coding or non- coding RNAs transcribed in the same orientation as the major transcription unit. Two operons associate protein coding genes and tRNA genes (*gbs0411-trmB*-*tRNA* Ser and *rplS-tRNA* Arg). Finally, 484 genes are transcribed as monocistronic units (Table 1). Globally, our operon map covers 85% of *S. agalactiae* coding genes and 98% of the genes expressed in the conditions we have tested (Additional file 5).

We also detected a significant level of antisense transcription (≥3 RPKM) on 138 genes (Additional file 6). For more than 70% of these genes, the antisense transcript was found to likely result from transcriptional read-through of terminators of genes in convergent orientation. These antisense transcripts, resulting from terminator read-through, extended up to 12 kb beyond the 3′ boundaries of their cognate CDS.

### Length distribution of 5′ untranslated regions (UTRs)

**Figure 4:**
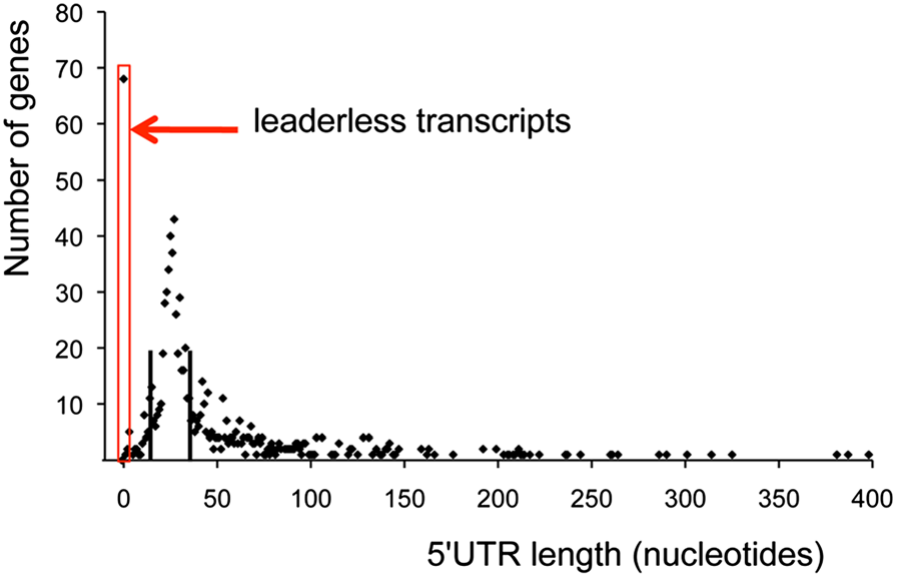
Distribution of 5′UTR lengths in *S. agalactiae.* Numbers of mRNA individual 5′UTRs according to their length based on 891 primary TSSs upstream coding genes. 78 genes were considered as leaderless with 5′UTRs shorter than 5 nucleotides, as indicated by the red box. 49% of the 5′UTRs have a size comprised between 15 and 35 nucleotides as indicated by black vertical lines

Analysis of the distance between TSSs and the translation initiating codons in *S. agalactiae* (Figure 4) showed that about half of the coding transcriptional units have 5′UTRs 15- to 35- nt long. Conversely, 8% of the mRNAs (n = 78) are leaderless, an intermediate proportion compared to *Helicobacter pylori* (1.5%) [20] and *Deinocococcus deserti* (60%) [39]. All of the leaderless transcripts share an AUG start codon and 90% of them begin at the A residue of the initiation codon. These two characteristics were found to be major determinants of ribosome recognition and binding to leaderless mRNAs in *E. coli* [40,41], suggesting efficient translation of leaderless mRNAs in *S. agalactiae*. Interestingly, the *holA/gbs0807* and *gbs0266* leaderless transcripts, encoding the DNA polymerase III subunit delta and a NAD-dependent dehydrogenase also showed an addition of pseudo-templated nucleotides at their 5′ ends and their translation might vary according to the amplitude of reiterative transcription. On the other hand, 84 transcription units, including genes involved in virulence such as the *cyl* (hemolysin) operon, the *scpB* gene encoding the C5A-peptidase, and the major TCS regulator of virulence *covRS* displayed 5′UTR longer than 100 nucleotides possibly involved in the regulation of transcript stability and translation.

### RNA-seq evidence for riboswitches and new cis-regulatory sequences

We combined different approaches to identify riboswitches and cis-regulatory sequences. First, long 5′UTRs (>75 nt) were screened for similarity to known cis-regulatory sequences in the Rfam database [42] and for the presence of predicted rho-independent terminators (Additional file 7). This *in silico* analysis was then confronted to experimental evidence: premature termination of transcription and production of a sRNA deduced from RNA-seq data. Altogether, 28 regulatory regions belonging to known Rfam families were detected (Table 1, Additional file 7), among which 24 were previously predicted through a computational analysis of the genome of *S. agalactiae* strain 2603 V/R [43]. For 19 of them, a non-coding RNA was detected together with a longer transcript encompassing the downstream CDS. Four additional 5′UTRs contained predicted rho-independent terminators, but transcription termination was not observed by RNA-seq analysis. The absence of termination likely results from a fully “open” conformation of the regulatory elements under the experimental growth conditions used. Therefore 23 out of the 28 Rfam-predicted 5′ cis-regulatory regions might act through a premature termination of transcription.

We identified riboswitches for flavin mononucleotide (FMN) (n = 2), for thiamine pyrophosphate (TPP) (n = 2), for purine (n = 1), for glycine (n = 1) and for prequeuosine (n = 2), as well as seven T-box leaders responsible for the response to uncharged tRNAs (Additional file 7). The other cis-regulatory elements included leader sequences acting through the binding of the ribosomal proteins L13, L19, L10, L20, L21 and binding sites for the regulator of pyrimidine biosynthesis PyrR. The ligands for certain sequences, such as one known as the yybP-ykoY leader found upstream the gene for a putative Ca2+ or Mg2+/ATPase (*gbs0560*) are still unknown. Interestingly, we observed that the efficiency of transcription termination directed by the yybP-ykoY leader increased in conditions of acid stress (Additional file 8). Indeed, while the expression of the leader sequence was not significantly modified after 20 minutes at pH 5.2, expression of the *gbs0560* CDS decreased by more than three-fold. This result suggests that, in *S. agalactiae,* the *yybP-ykoY* leader riboswitch senses directly or indirectly a pH modification. A function of pH-responsive riboregulator was also recently proposed for the *srfA* riboswitch in *E. coli*, the sequence of which partly overlaps with the yybP-ykoY leader sequence consensus [44]. An inverse behavior was observed for the glycine riboswitch upstream *gbs1212* encoding a putative alanine/glycine cation symporter. While transcription predominantly terminated at the rho-independent terminator at neutral pH, the shift to a lower pH was associated with an increased transcription of the coding part, suggesting a change in the riboswitch structure. This glycine riboswitch might therefore act as a dual riboswitch sensing both the presence of glycine and another signal induced during acid stress (Additional file 8).

Eleven sequences located in the 5′UTR of coding genes share characteristics with cis-regulatory elements, despite the absence of similarity with Rfam families (Table 1 and Additional file 7). For all these sequences, RNA-seq experiments revealed a short non-coding (ncRNA) form and a long transcript sharing the same TSS, supporting a cis-regulatory mechanism. We identified one of these sequences in the 5′UTR of the essential operon *gbs0413-gbs0418* encoding the ribosome maturation factor RimP, the elongation factor NusA, the translation initiation factor IF2, the ribosome-binding factor A and two conserved proteins of unknown functions. Transcription termination occurred 31 nucleotides downstream the TSS at a predicted intrinsic terminator. NusA has been shown to stimulate pausing of the elongating RNA polymerase, facilitating transcription termination at rho-independent terminators [45,46]. Combined with our data, this suggests that NusA mediates in *S. agalactiae* a negative feedback regulation of the *gbs0413-gbs0418* operon. A similar mechanism has been proposed from the analysis of *nusA* mutations in *E. coli* for the *metY-nusA-infB* operon [47]. Therefore, this autoregulation might be conserved among the gram-negative and gram-positive bacteria.

**Figure 5:**
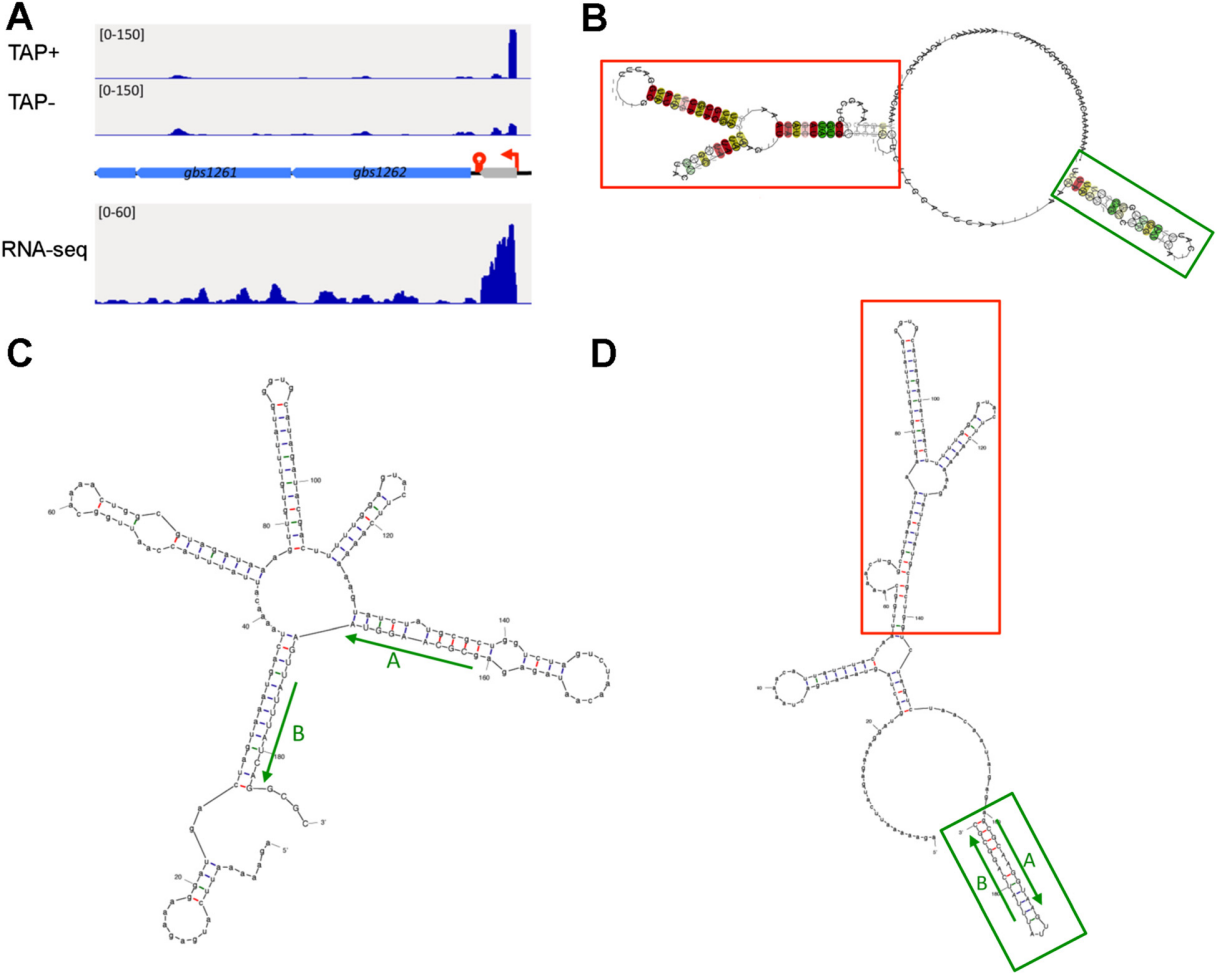
Identification of a novel riboswitch upstream *gbs1262.* **A** *.* Transcriptional organization deduced from dRNA-seq and RNA-seq experiments. Reads aligning upstream *gbs1262* were visualized by IGV browser [67]. The sequence of the sRNA resulting from transcription premature arrest at a rho-independent terminator is indicated as a grey arrow. **B**. Secondary structure predicted by RNalifold [71], based on the alignment of 14 sequences similar to *gbs1262* 5′UTR in Lactobacillales and upstream a potential tryptophan-related gene in *F. nucleatum* (Additional file 9). The two conserved folded structures are indicated in red and green boxes, with the green box corresponding to a rho-independent terminator. Sequence covariations supporting the consensus structure are marked by color: red marks pairs with no sequence variation; ochre and green mark pairs with 2 or 3 types of pairs, respectively. **C** and **D**. Alternative structures of *gbs1262* putative riboswitch as determined by mfold [72], showing the formation of an antiterminator structure*.*

Two potential cis-regulatory elements, 195- and 270-nt long, respectively, are located upstream two paralogous operons encoding ABC-transporters, *gbs1262-gbs1260* and *gbs2033-gbs2031,* respectively, hinting that transcription of these operons might be regulated by new riboswitches sensing the substrates of the ABC-transporters. Searching for similar sequences revealed that the two elements are conserved among Lactobacillales (Additional file 9 and Additional file 10). A secondary structure based on sequence alignment and sequence covariations was predicted for each element (Figure 5 and Additional file 11). In addition to genes encoding ABC transporters, the *gbs2033* riboswitch was also identified upstream the *Enterococcus faecium aroFBC-tyrA* operon involved in aromatic amino acid biosynthesis. A sequence similar to *gbs1262* riboswitch was identified upstream the gene encoding a tryptophan synthase beta chain in *Fusobacterim nucleatum*. These results suggest that these cis-regulatory elements belong to new families of riboswitches sensing aromatic amino acids or their precursors.

### Genome-wide identification of *S. agalactiae* sRNA repertoire

TSS mapping identified 39 TSSs potentially initiating an antisense transcript, as well as 44 TSSs in intergenic regions not directly linked to protein coding genes or to tRNA genes. The RNA-seq data confirmed transcription of these 73 antisense or intergenic sRNA and identified three supplementary sRNAs: two located in intergenic regions, which likely result from the processing of a longer RNA, as no TSS was identified by dRNA-seq experiments and one sRNA initiated at a TSS located at the end of a non-expressed coding gene (Table 1 and Additional file 12). 11 among the 86 sRNAs detected were predicted in a previous *in silico* study that identified 41 sRNAs in intergenic regions and 99 antisense RNAs [48]. The incomplete overlap between sRNAs identified through bioinformatics and experimental detection has already been noticed [24] and may be due to sRNAs expressed under very specific conditions. On the other side, *in silico* approaches may generate false positive candidates or incomplete predictions linked to the criteria used. Some of the antisense RNAs, such as those overlapping *sfbA* [49] or the gene for the streptococcal histidine-triad family protein (*gbs1306*) [50], might be involved in the regulation of *S. agalactiae* virulence by interfering with the stability or translation of their cognate mRNA.

Seven sRNAs (Snr015/csRNA10, Srn024/csRNA11, Srn070/csRNA12, Srn008, Srn017, Srn046/*asd* and Srn073) were chosen for further expression analysis by Northern blot (Additional file 13 A). These experiments confirmed that all are expressed and showed differential patterns of growth phase-dependent accumulation and induction in response to stresses. For example, Srn073 was induced at the onset of the stationary phase (Additional file 13 A) and during stress adaptation to high NaCl concentration (Additional file 13). In contrast expression of Srn046, a 200 nt-long RNA with a Rfam asd RNA motif [51] also identified in *Streptococcus pyogenes* [52], was strongly down-regulated at late stationary phase (Additional file 13 A).

**Figure 6:**
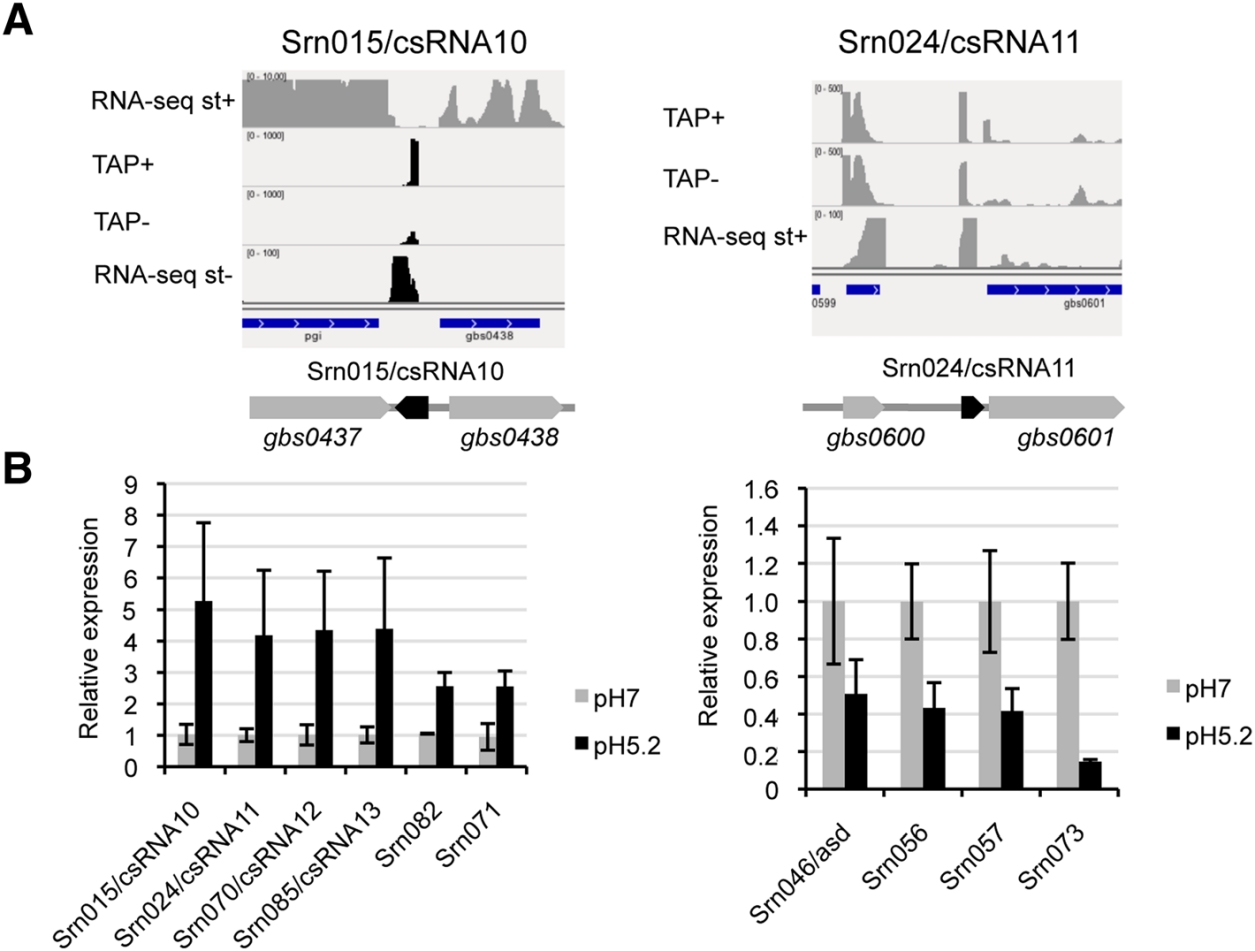
Modifications in sRNA expression in response to an acidic stress. **A**. Detection of Srn015/csRNA10 and Srn070/csRNA12 by combined analysis of RNA-seq and dRNA-seq experiments is given as an example of sRNA detection **B**. sRNA expression was quantified using RNA-seq (triplicate experiments) on exponentially growing bacteria submitted or not to a 20 min-acid stress. Normalization and statistical assessment were performed using the EdgeR software. Expression values were arbitrarily fixed to 1 for samples grown at pH7. Ten sRNAs were shown differentially regulated (p <0.05 after correction for multiple-testing adjustment) in acidic conditions: 6 up-regulated, left panel and 4 down-regulated, right panel.

Cia-dependent sRNAs (csRNAs) were first described in *Streptococcus pneumoniae* and *Streptococcus mutans* where they are involved in stationary-phase autolysis [53], regulation of competence [54] and virulence [55]. Based on sequence similarity, four csRNAS were predicted in *S. agalactiae* [56]. Here, we detected by RNA-seq these four csRNAs, showed that their sizes ranged from 60 to 140 nt and identified their respective TSSs and promoter sequences (Additional file 12). The TSSs could not be detected in dRNA-seq experiments with the ∆*ciaRH* mutant, in agreement with a strong control of their expression by CiaR (Additional file 2). Northern blot analysis on csRNA10/Srn015, csRNA11/Srn024 and csRNA12/Srn070 showed growth-phase dependent accumulation (Additional file 13 A). In addition, Srn015 and Srn024 were strongly induced in response to acid stress (Additional file 13 A). This induction was confirmed by quantitative analysis of the RNA-seq experiments (Figure 6). The expression of the 4 csRNAs and of 2 additional sRNAs (Srn082, Srn071) was induced by more than two-fold at a pH of 5.2, whereas four others (Srn046/asd, Srn056, Srn057 and Srn073) were downregulated. These observations strongly suggest that sRNA-mediated regulation plays a role in *S. agalactiae* adaptation to the various environments it encounters during colonization and infection, particularly to acidic conditions. Quantitative analysis of the RNA-seq data also revealed more than two-fold variations in the expression of 284 coding genes as a result of pH modification (Additional file 14). In particular, many genes involved in *S. agalactiae* virulence were expressed at a higher level at neutral pH, such as the *cyl* operon and the capsule operon and the genes encoding the C5A peptidase, the C5A-related peptidase and the fibrinogen-binding proteins A, B and C, in agreement with previously published results [18]. Significant variations in the expression of four sRNAs (csRNA10, csRNA11, Srn073 and Srn073) and the *cylE* gene in response to acid stress were further confirmed by using qRT-PCR experiments (Additional file 13 B).

In addition to the four csRNAs, 10 other sRNAs matched Rfam families, including transfer-messenger RNA (tmRNA/SsrA) involved in the recycling of stalled ribosomes, 6S/SsrS RNA, signal recognition particle RNA (SRP/4.5S) and M1/RnpB RNA, the RNA component of RibonucleaseP (RNaseP) involved in the maturation of tRNAs and of some sRNAs. Combined TSS mapping and RNA-seq revealed that RnpB RNA likely results from the processing of a longer transcript, comprising the CDS for the cell division protein GpsB (*gbs0291*), for a RNA methylase potentially involved in the modification of 23S RNA m2G2445 (*gbs0292*) and for a protein of unknown function (*gbs0293).* The association between the three coding genes and RNaseP mRNA is conserved among all streptococci, suggesting a functional and regulatory link between these genes. Recently, RNaseJ was shown to be implicated in generating *rnpB* 5′ end in *Staphylococcus aureus* [57].

The tracrRNAs are key components of type II clustered regularly interspaced short palindromic repeats (CRISPR) system. As first shown in *S. pyogenes,* they direct the processing of crRNAs involved in the sequence-specific recognition and the silencing of invading mobile elements [58]. Two tracrRNA forms, with the same 3′ end but differing by their TSS, were identified, as well as 15 small crRNAs resulting from the efficient processing of a 1,069 nt-long pre-crRNA in strain NEM316. The leader-proximal and leader-distal crRNAs were more abundant than crRNAs from the central part of the array, suggesting differential maturation or stability of the crRNAs of the array (Additional file 15). Analysis of RNA 5′ ends allowed the characterization of crRNA maturation sites at the nucleotide level, showing that crRNAs resulted from processing at two sites, as previously observed in *S. pyogenes* [58] (Additional file 15). Unique to *S. agalactiae*, a short ~100 nt-long antisense RNA transcript, overlapping the tracrRNA 3′ end, was detected and might indirectly regulate the CRISPR response by modulating expression of the tracrRNAs (Additional file 15). This supplementary level of complexity in the regulation of crRNA maturation might help *S. agalactiae* to cope with the various genome invaders encountered in the different niches it colonizes [59].

## Conclusions

In this study, we combined dRNA-seq and strand-specific RNA-seq to characterize, at the single nucleotide resolution, the transcriptional landscape of *S. agalactiae*. With our modified protocol of dRNA-seq analysis, we were able to detect a surprising high level of reiterative transcription in *S. agalactiae.* To our knowledge, this is the first genome-wide determination of the genes affected by reiterative transcription. Initiation of transcription is a target of global regulations such as these, which are mediated by the intracellular concentration of initiating nucleotides and by the stringent response. In this context, repetitive nucleotide addition might serve as a mechanism to translate this global information on the metabolic status of the cell into modifications in gene expression. This might be accomplished by affecting the transition to the transcription elongation step or by leading to changes in the secondary structure of the transcript. Based on this work, systemic mining of RNA-seq data will likely uncover the extent of reiterative transcription in other prokaryotes or eukaryotes and reveal new mechanisms of regulation relying on the non templated addition of nucleotides at the 5′-end of mRNAs. Combined RNA-seq and dRNA-seq approaches were also efficient in characterizing new cis-regulatory regions, riboswitches and sRNAs. By introducing this new information into the quantitative analysis of our RNA-seq data, we analyzed variations in sRNA levels in response to acid stress. We found that expression of at least ten sRNAs, including the four csRNAs, is modified in response to a pH variation, suggesting that these sRNAs are involved in *S. agalactiae* adaptation to its changing environments. Since adaptation to acidic conditions is important to colonize the vaginal niche and to survive in macrophage phagosome, these sRNAs might play a role in controlling the virulence of *S. agalactiae*, as previously shown for one of the csRNAs in *S. pneumoniae* [55]. Our results pave the way for deciphering the regulatory networks in which these sRNAs are involved and further understanding their biological functions.

## Methods

### Bacterial strains and growth conditions

All experiments were conducted in the *S. agalactiae* strain NEM316 [11]. The NEM316 mutant derivatives used in this work are described in Additional file 16. In frame deletion mutants of the *ciaRH* and *relRS* genes were constructed by using splicing-by-overlap-extension PCR, as previously described [60] and the oligonucleotide pairs *ciaR*_Eco-*ciaR*_Int1 and *ciaH*_Int2-*ciaH*_Bam, or *relR*_Eco-*relR*_int1 and *relH*_int2-*relH*_Bam, respectively (Additional file 17). Bacteria were cultured at 37°C in Todd Hewitt (TH) medium (Difco) to mid-exponential growth phase (OD_600_ = 0.3-0.4) (MidE), late exponential phase (OD_600_ = 1–1.2) (LE) or stationary (OD_600_ = 1.6-1.8) phase (ST). The strain NEM316 was also grown to OD_600_ = 0.3-0.4 in RPMI medium, without phenol red, supplemented by 50 mM Hepes, pH 7 and 1% glucose. This OD_600_ corresponded to early stationary growth phase as, in this culture medium, the growth plateau was reached at OD_600_ = 0.35-0.40. For RNA-seq and dRNA-seq experiments assessing the effects of acid stress, strain NEM316 was grown to OD_600_ = 0.4 in TH medium, centrifuged and resuspended in fresh TH medium adjusted or not to pH 5.2 and further incubated for 20 additional minutes at 37°C.

### RNA extraction and mRNA enrichment

Total RNA was prepared as previously described [14]. Residual DNA was removed with TURBO DNAse (Ambion). RNA integrity was verified with the Agilent Bioanalyzer 2100. Only RNA preparations with RNA Integrity Numbers greater than 9 were kept for analyses. mRNA enrichment was performed with the MICROBExpress Kit (Ambion). Depletion of 16S and 23S ribosomal RNAs was confirmed with the Agilent Bioanalyzer 2100.

### dRNA-seq library preparation and sequencing

Depleted RNA samples were divided into two subsamples (hereby TAP+ and TAP-) containing the equivalent of 7.5 μg of total RNA and used for dRNA-seq library preparation as described [61]. The TAP+ subsample was pretreated with 10 U of Tobacco Acid Phosphatase (Tebu-Bio), the other steps being strictly run in parallel for the two subsamples. In brief, TAP treatment was followed by ligation with an excess of 5′ adapter (Illumina TruSeq Small RNA kit) and by reverse transcription using a random primer (RPO primer: 5′CCTTGGCACCCGAGAATTCCANNNNNN-3′). The first strand cDNA/RNA hybrids were then run on a 2% Low Range Agarose (Biorad). cDNAs ranging from 120 to 250 bp were extracted from a gel slice by using the Qiaquick gel extraction kit (Qiagen) and PCR amplified for 14 cycles using the Illumina primer RP1, and one of the indexed primers (Illumina TruSeq Small RNA kit). The resulting PCR products were purified with Agencourt AMPure Beads XP (Beckman) and sequenced on the Illumina GAIIX or HiSeq 2000.

### Strand-specific RNA-seq library preparation

Strand-specific RNA-seq libraries were generated with the Illumina primer ligation method, as described [62] with the modifications described in [61]. In brief, after depletion of ribosomal RNAs using the MICROBExpress Kit (Ambion), RNA was treated with TAP (Tebu-Bio), fragmented using the RNA fragmentation Reagent kit (Ambion). Dephosphorylation of the fragmented RNA by using the antarctic phosphatase (Biolabs), and rephosphorylation with the T4 polynucleotide kinase (Biolabs) were followed by a purification step through RNeasy MinElute Cleanup columns (Qiagen). The RNA was then successively ligated to the 3′ RNA adapter (Illumina TruSeq Small RNA kit) by using truncated T4 RNA ligase 2 (Biolabs) and to the 5′ RNA adapter (Illumina TruSeq Small RNA kit) with T4 RNA ligase (Biolabs). RNA ligated to the two adapters was reverse-transcribed with the Superscript II Reverse Transcriptase (Life Technologies) and the RNA reverse transcription primer (Illumina TruSeq Small RNA kit). The resulting cDNAs were amplified for 13 PCR cycles with Phusion Taq polymerase using the RP1 primer and one of the indexed PCR primers (Illumina TruSeq Small RNA kit).

### Data availability

The raw dRNA-seq and RNA-seq reads are available in the ArrayExpress database (www.ebi.ac.uk/arrayexpress) under accession number: E-MTAB-3200.

### Read mapping and alignment to the reference genome

Sequencing reads generated from dRNA-seq and RNA-seq libraries were trimmed for adapter sequences with Cutadapt [63] and reads shorter than 25 nucleotides were discarded. Mapping was performed on *S. agalactiae* NEM316 genome sequence (NC_004368.1) by the software Bowtie (version 0.12.7) [64] with a seed of 25 nt and allowing two mismatches. Reads that mapped at more than four different positions on the genome were discarded, i. e. reads corresponding to rRNA. To characterize reads corresponding to transcripts with pseudo-templated nucleotides on their 5′ ends, reads were first aligned by Bowtie with a seed of 25 nt and allowing no mismatches. In reads that did not align under these conditions, we trimmed 10 nucleotides at the 5′ end and aligned them a second time to the NEM316 genome with Bowtie. Reads recovered under these conditions were further characterized for their content in pseudo-templated nucleotides by a custom Python program.

### Statistical assignment of TSSs and promoter sequence annotation

For each dRNA-seq experiment, the positions of the 5′ ends of the reads were recorded and the numbers of reads under TAP+ and TAP- conditions at each position were counted. Positions where the sum of reads in TAP+ and TAP- conditions was less than six were not further considered. A statistical assessment of TSS positions was performed by EdgeR (version 3.2.4) [65] for each dRNA-seq experiment, as follows: after normalization for the size of the TAP+ and TAP- libraries, a common dispersion was estimated assuming that the TSS positions represented only a small fraction of the total positions. This common dispersion value was used with the exact test to generate a list of positions for which reads were statistically more abundant in the TAP+ library. p-values after multiple testing adjustment procedure (Benjamini Hochberg) [66] were calculated but found to be generally too stringent, eliminating many potential TSSs. Therefore, a position was considered to correspond to a TSS with a high-level of confidence either if it was found with a p-value ≤ 0.05 after multiple testing adjustment procedure or if it was found in more than two dRNA-seq experiments with a simple p-value ≤ 0.05. For multiple genomic positions located within less than 5 bases, the position with the highest number of supporting reads was selected as the TSS. Potential TSSs detected in only one dRNA-seq experiment and with a p-value ≥ 0.05 after multiple testing adjustment procedure were visually inspected with the IGV genome browser [67] and kept only if they were located in an intergenic region and clearly corresponded to a transcript 5′-end identified by RNA-seq. The 50 nt upstream each TSS were extracted from the NEM316 genome sequence and searched by MEME (http://meme.nbcr.net/meme/tools/meme) and custom Python scripts for promoter motifs.

### RNA-seq data analysis

Reads generated from strand-specific RNA-seq experiments were aligned to the genome of strain NEM316. The number of reads mapping to each predicted CDS was determined by Rsamtools (version 1.13.35), GenomicRanges (version 1.13.39) and GenomicFeatures (version 1.12.3) in R 3.0.1. The gene expression values were quantified in terms of reads per million (RPKM) defined as the total number of reads mapping to the feature divided by feature length (in kbp) normalized by the total number of reads (in millions) [68]. For differential expression analysis, normalization and statistical analyses were performed using the EdgeR Software (version 3.2.4) [65], p-values were adjusted for multiple testing using the false discovery rate controlling procedure [66].

### Determination of operon structure and mapping of transcript 3′ ends

To map transcript 3′ ends, the coverage per nucleotide was determined along both strands of the genome sequence by using the SAMtools [69] (version 0.1.12a) and variations in coverage were calculated with a custom R script. A more-than-five-fold decrease in coverage among two intergenic positions distant by less than 10 nucleotides was considered as indicative of a major transcript 3′ end. Only positions covered by at least five reads were taken into consideration. When several clustered positions fulfilled this condition, only the most downstream was considered as the mRNA end. Termination was considered to be complete when a region not covered by any read was found immediately after the transcript end. Identification of transcript ends was performed using six RNA-seq libraries and only transcript ends identified from at least two RNA-seq experiments and mapping in an intergenic region were considered. The list of the transcript ends was compared with the positions of potential terminators as described by de Hoon *et al.* [38]. Supplementary terminators were searched with ARNold (http://rna.igmors.u-psud.fr/toolbox/arnold/) or TranstermHP (http://transterm.cbcb.umd.edu/). Two consecutive genes in the same orientation were considered as belonging to different transcription units (TU) if transcription of the first gene ends with a 100% efficient termination site or if a primary TSS was detected upstream the second gene. Accordingly, TU were classified into one of three categories: 1) monocistronic TU; 2) simple operons, composed of several genes preceded by a primary TSS and separated from the next TU by a 100% efficient termination site and/or a primary TSS; 3) composite operons preceded by a primary TSS and characterized by the presence of at least one internal TSS and/or one inefficient terminator leading to variations in gene expression levels along the operon.

### Northern blot validation of sRNAS

RNA were extracted from *S. agalactiae* NEM316 strain grown in TH medium supplemented with yeast extract, 2 g/l (THY) at 37°C. Samples were harvested at OD_600_ = 0.3, 0.6, 1.2, 2 and late stationary phase (overnight culture). To study the effect of stress conditions, the strain NEM316 was grown in THY medium. At OD_600_ = 0.6, the medium was either supplemented with lysozyme (200 ng/ml) or bacteria were centrifuged and resuspended in fresh THY adjusted to pH 4.5, or supplemented with 1 M NaCl or with 4 mM H_2_0_2_, final concentrations. Extraction of total RNA and Northern blot hybridization with α^32^-P 3′-labeled oligonucleotides (described in Additional file 17) were performed as previously described [70].

## Additional files

Additional file 1:
**Number of reads mapped in the 22 dRNAseq and RNAseq experiments.**
Additional file 2:
**Characteristics of promoter sequences.**
Additional file 3:
**Regulation of** ***pyrG*** **transcription through reiterative transcription.** A*. pyrG* transcripts encoding CTP synthetase were found by dRNA-seq to initiate at a C or a G residue (non template strand) shown in red. B. Initiation at the G residue led to reiterative transcription and incorporation of pseudo-templated G nucleotides at the transcript 5′ end (in green). C. Alternative structures that form in the absence or presence of pseudo-templated nucleotides. The incorporation of pseudo-templated G residues prevents transcription attenuation by allowing an antiterminator hairpin structure. RNA-seq experiments show that in exponential growth, both a sRNA terminating at the predicted terminator and a long transcript including *pyrG* were detected (Additional file 7). Additional file 4:
**Detection of transcript ends and comparison with** ***in silico*** **predicted** **rho-independent** **terminators.**
Additional file 5:
**Operon organization of the NEM316 genome.**
Additional file 6:
**Genes covered by high levels (>3 RPKM) of antisense transcripts in conditions of** **mid-exponential** **growth.**
Additional file 7:
**Characteristics of potential 5′cis-regulatory sequences in** ***S.agalactiae*** **(strain NEM316) as detected by dRNAseq and RNAseq analyses.**
Additional file 8:
**Acid stress modifies transcription termination mediated by yybP/ykoY leader and** **glycine-riboswitch.** A. The yybP/ykoY leader located upstream *gbs0560* encoding a cation-transporting P-ATPase. B. The glycine-riboswitch upstream *gbs1212* encoding a putative amino acid transporter. Left panels: dRNA-seq (TAP+ and TAP- samples) and RNA-seq data from exponentially growing bacteria submitted or not to a 20 min-acid stress (pH5.2) are visualized with the IGV Genome Browser. Right panels: Transcription levels of the riboswitch and of the downstream genes under both conditions. Expression was quantified using EdgeR. Transcription levels of the two sRNA did not significantly vary between the two conditions tested. In contrast, expression of the downstream genes was significantly down-regulated *(gbs0560)* or up-regulated *(gbs1212)* (p < 0.01) in conditions of acid stress revealing modifications in the efficiency of transcription termination directed by the two riboswitches. Means of triplicates ± SD. Additional file 9:
**Alignment of DNA sequences similar to** ***gbs1262*** **5′UTR.** The DNA sequences in 14 Lactobacillales and upstream a potential tryptophan-related gene in *F. nucleatum* were extracted from Genbank. Accession numbers are given in the lower panel. Alignment was performed by using clustalW and a secondary structure was calculated with RNAalifold. In the alignment, sequence covariations supporting the consensus structure are marked by color: red marks pairs with no sequence variation; ochre and green mark pairs with 2 or 3 types of pairs, respectively. Additional file 10:
**Alignment of DNA sequences similar to** ***gbs2033*** **5′UTR.** The DNA sequences in ten Lactobacillales and upstream the *aroF* gene in *E. faecium* were extracted from Genbank. Accession numbers are given in the lower panel. Alignment was performed by using clustalW and a secondary structure was calculated with RNAalifold. In the alignment, sequence covariations supporting the consensus structure are marked by color: red marks pairs with no sequence variation; ochre and green mark pairs with 2 or 3 types of pairs, respectively. Additional file 11:
**Identification of a novel riboswitch upstream** ***gbs2033.*** A. Identification from dRNA-seq and RNA-seq experiments. Reads aligning upstream *gbs2033* were visualized by the IGV browser. The sequence of the sRNA resulting from transcription premature arrest at a rho-independent terminator is indicated as a grey arrow. B. Structure prediction by RNalifold, based on the alignment of 9 sequences similar to *gbs2033* 5′UTR in Lactobacillales and upstream the *aroF* gene in *E. faecium* (Additional file 10)*.* The two folded and conserved structures predicted are indicated in red and blue boxes. C and D. Two alternative structures o*f gbs2033* putative riboswitch as determined by mfold. The two regions found by Rnalifold are indicated in red and blue boxes. The green box corresponds to a rho-independent terminator. Additional file 12:
**List of ncRNAs identified by** **RNA-sequencing** **(not including sRNAs generated from** **cis-regulatory** **regions).**
Additional file 13:
**Growth phase and** **stress-dependent** **expression of NEM316 sRNAs.** A. Northern blot experiments showing the expression of seven ncRNAs (Snr015/csRNA10, Srn024/csRNA11, Srn070/csRNA12, Srn008, Srn017, Srn046/*asd* and Srn073) according to growth phase conditions or in response to various stresses. Total RNAs were prepared from cultures harvested at OD_600_: 0.3, 0.6, 1.2, 2 and late stationary phase (STAT) or grown to O.D_600_ = 0.6 and subjected to 15 min lysozyme (200 ng/ml), 30 min acid (pH 4.5), 20 min salt (NaCl 1 M) or 15 min oxidative (H_2_0_2_ 4 mM) stresses. 5S RNA and tmRNA were used as loading controls. B. qRT-PCR experiments on four sRNAs (csRNA10, csRNA11, Srn073, Srn071) and the *cylE* gene. mRNAs were extracted from triplicate cultures of *S. agalactiae* bacteria grown in TH medium. Bacteria were harvested at O.D_600_ = 0.4 and resuspended in fresh TH adjusted or not to pH 5.0. Real-time PCR was performed on cDNA preparations using the SYBR green detection system (Applied Biosystems, Warrington, UK). Primers are listed in Additional file 17. Mean ± SD (N = 3). A p-value < 0.05 was considered as significant (unpaired bilateral Student’s *t* test). Additional file 14:
**Genes regulated more than** **2-fold** **in** ***S. agalactiae*** **strain NEM316 after a 20 min-exposure to pH 5.2 versus pH 7.0.**
Additional file 15:
**Transcriptional organization of the CRISPR locus in** ***S. agalactiae*** **strain NEM316.** A. Transcription and maturation profiles of the crRNA and tracrRNA. Combined results from the RNA-seq and dRNA-seq experiments allowed to define the TSS for the *cas* operon, for the precrRNA and for the two tracrRNAs as well as to characterize the maturation profile of the CRISPR array. In addition, they revealed a novel sRNA (Srn036) indicated by a red arrow, partially overlapping tracrRNA, indicated by blue arrows, in antisense orientation that might interfere with tracrRNA functions or regulation. B. Position of the maturation sites in crRNA. The large blue arrow indicates the maturation site resulting from hybridization with the tracrRNA and RNaseIII digestion, whereas the thin green arrow indicates the position of the second processing site of unknown origin. Additional file 16:
**Bacterial strains used in the study.**
Additional file 17:
**Oligonucleotides used in this study.**

## Abbreviations

TSS: Transcription start site
RNA-seq: RNA-sequencing
dRNA-seq: Differential RNA-sequencing
UTR: Untranslated region
TAP: Tobacco acid pyrophosphatase
TH medium: Todd-Hewitt culture medium
WT: Wild-type
CDS: Coding sequence
RPKM: Reads per kilobase per million mapped reads
