# Supplementary material for: Single nucleotide resolution RNA-seq uncovers new regulatory mechanisms in the opportunistic pathogen Streptococcus agalactiae
Source: BMC Genomics. 2015 May 30;16(1):419. doi: 10.1186/s12864-015-1583-4 (PMC4448216; doi:10.1186/s12864-015-1583-4)
Supplement: Additional file 1: — Number of reads mapped in the 22 dRNAseq and RNAseq experiments. [file 12864_2015_1583_MOESM1_ESM.pdf]

Additional file 1: **Number of reads mapped in the 22 dRNAseq and RNAseq experiments**

Strain	Condition	Sample	Number of reads after adapter trimming	Mapped reads	Number of reads mapped to non rRNA regions
wt	mix[1] (MG)	TAP+	12.1 M	11.7 M	4.7 M
wt	mix[1] (MG)	TAP-	10.4 M	10.0 M	2.5 M
wt	Late exponential (LE)	TAP+	14.5 M	12.6 M	2.4 M
wt	Late exponential (LE)	TAP-	14 M	11.7 M	1.1 M
wt	mid-exponential (midE)	TAP+	22.9 M	21.9 M	7.6 M
wt	mid-exponential (midE)	TAP-	16.2 M	15.4 M	2.2 M
wt	mid-exponential, 20 min ph5.2 (AcSt)	TAP+	6.5 M	6.2 M	2.6 M
wt	mid-exponential, 20 min ph5.2 (AcSt)	TAP-	5.3 M	5.0 M	0.9 M
Δ*ciaRH*	Late exponential (ΔciaRHLE)	TAP+	11.8 M	11.6 M	6.5 M
Δ*ciaRH*	Late exponential (ΔciaRHLE)	TAP-	17.9 M	17.4 M	2.7 M
Δ*covRS*	mid-exponential (ΔcovRSmidE)	TAP+	13.5 M	13.2 M	4.3 M
Δ*covRS*	mid-exponential (ΔcovRSmidE)	TAP-	15.0 M	14.6 M	2.4 M
Δ*covRS*	Late exponential (ΔcovRSLE)	TAP+	11.7 M	11.3 M	3.4 M
Δ*covRS*	Late exponential (ΔcovRSLE)	TAP-	8.7 M	8.6 M	2.1 M
Δ*relRS*	mid-exponential (ΔrelRSmidE)	TAP+	19.9 M	19.5 M	5.7 M
Δ*relRS*	mid-exponential (ΔrelRSmidE)	TAP-	25.6 M	24.9 M	4.4 M
wt	mid-exponential (midE)	RNAseq	15.9 M	14.6 M	5.7 M
wt	mid-exponential (midE)	RNAseq	13.0 M	11.8 M	5.4 M
wt	mid-exponential (midE)	RNAseq	13.5 M	12.5 M	5.5 M
wt	mid-exponential, 20 min pH5.2 (AcSt)	RNAseq	17.3 M	15.8 M	7.8 M
wt	mid-exponential, 20 min pH5.2 (AcSt)	RNAseq	21.5 M	19.2 M	8.8 M
wt	mid-exponential, 20 min pH5.2 (AcSt)	RNAseq	15.4 M	14.0 M	6.8 M

[1]: mix relates to a mixture of RNAs prepared in rich culture medium (TH) in exponential and stationary growth phases and in minimal culture medium (RPMI) at the end of exponential phase
