# Supplementary figures and images for: Single nucleotide resolution RNA-seq uncovers new regulatory mechanisms in the opportunistic pathogen Streptococcus agalactiae
Source: BMC Genomics. 2015 May 30;16(1):419. doi: 10.1186/s12864-015-1583-4 (PMC4448216; doi:10.1186/s12864-015-1583-4)

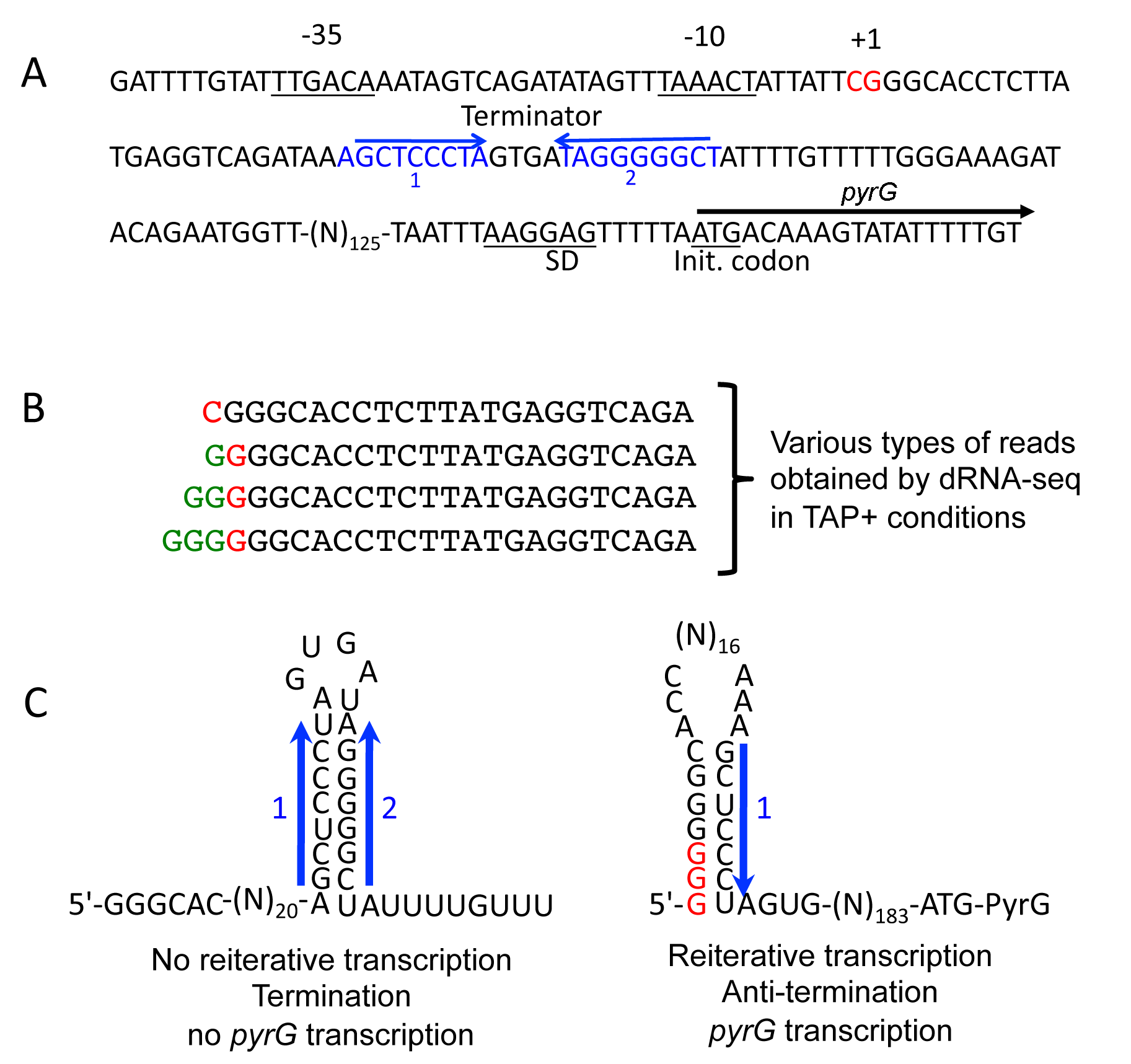

Supplement: Additional file 3: — Regulation of pyrG transcription through reiterative transcription. A. pyrG transcripts encoding CTP synthetase were found by dRNA-seq to initiate at a C or a G residue (non template strand) shown in red. B. Initiation at the G residue led to reiterative transcription and incorporation of pseudo-templated G nucleotides at the transcript 5′ end (in green). C. Alternative structures that form in the absence or presence of pseudo-templated nucleotides. The incorporation of pseudo-templated G residues prevents transcription attenuation by allowing an antiterminator hairpin structure. RNA-seq experiments show that in exponential growth, both a sRNA terminating at the predicted terminator and a long transcript including pyrG were detected (Additional file 7). [file 12864_2015_1583_MOESM3_ESM.tiff]

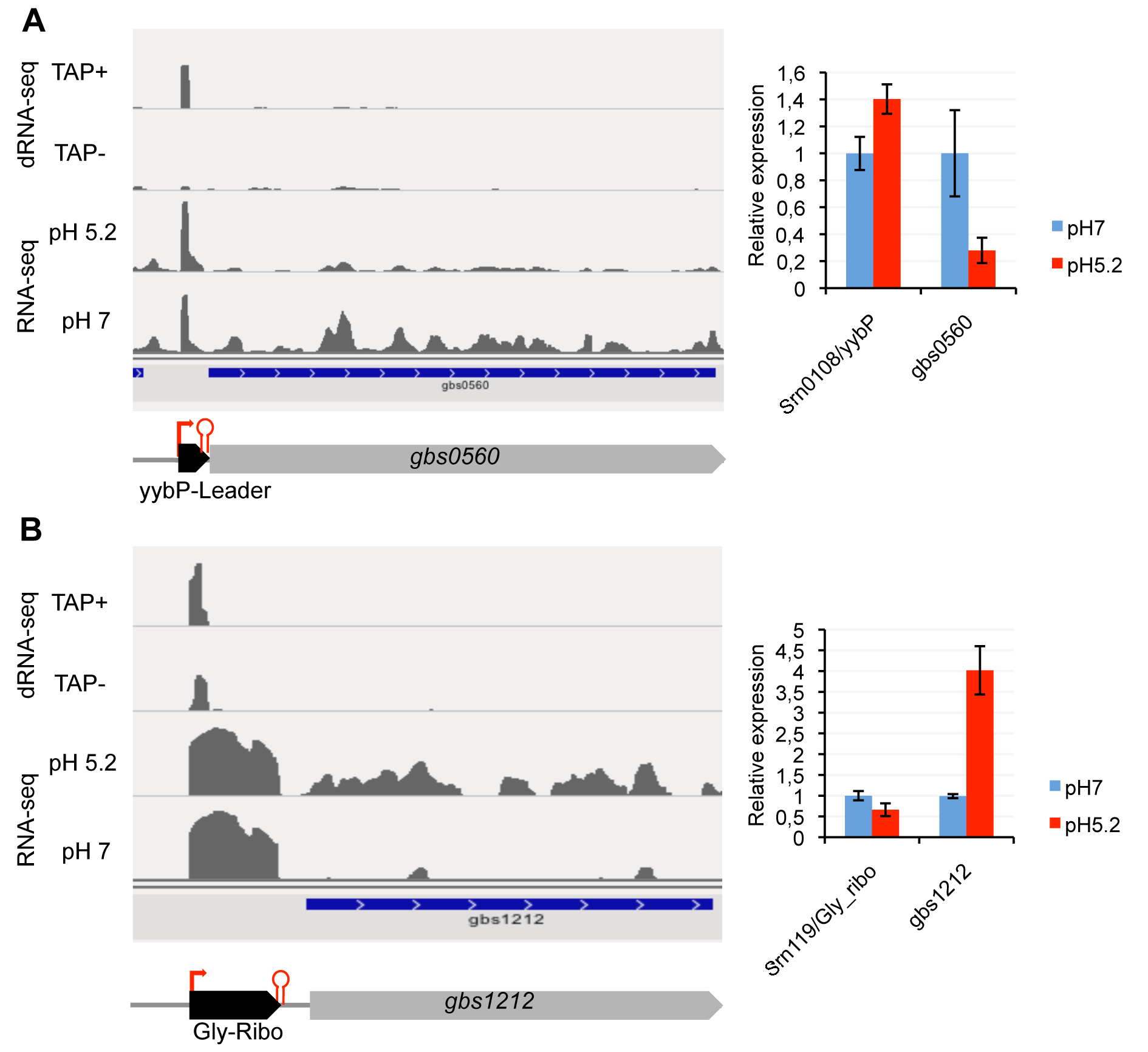

Supplement: Additional file 8: — Acid stress modifies transcription termination mediated by yybP/ykoY leader and glycine-riboswitch. A. The yybP/ykoY leader located upstream gbs0560 encoding a cation-transporting P-ATPase. B. The glycine-riboswitch upstream gbs1212 encoding a putative amino acid transporter. Left panels: dRNA-seq (TAP+ and TAP- samples) and RNA-seq data from exponentially growing bacteria submitted or not to a 20 min-acid stress (pH5.2) are visualized with the IGV Genome Browser. Right panels: Transcription levels of the riboswitch and of the downstream genes under both conditions. Expression was quantified using EdgeR. Transcription levels of the two sRNA did not significantly vary between the two conditions tested. In contrast, expression of the downstream genes was significantly down-regulated (gbs0560) or up-regulated (gbs1212) (p < 0.01) in conditions of acid stress revealing modifications in the efficiency of transcription termination directed by the two riboswitches. Means of triplicates ± SD. [file 12864_2015_1583_MOESM8_ESM.tiff]

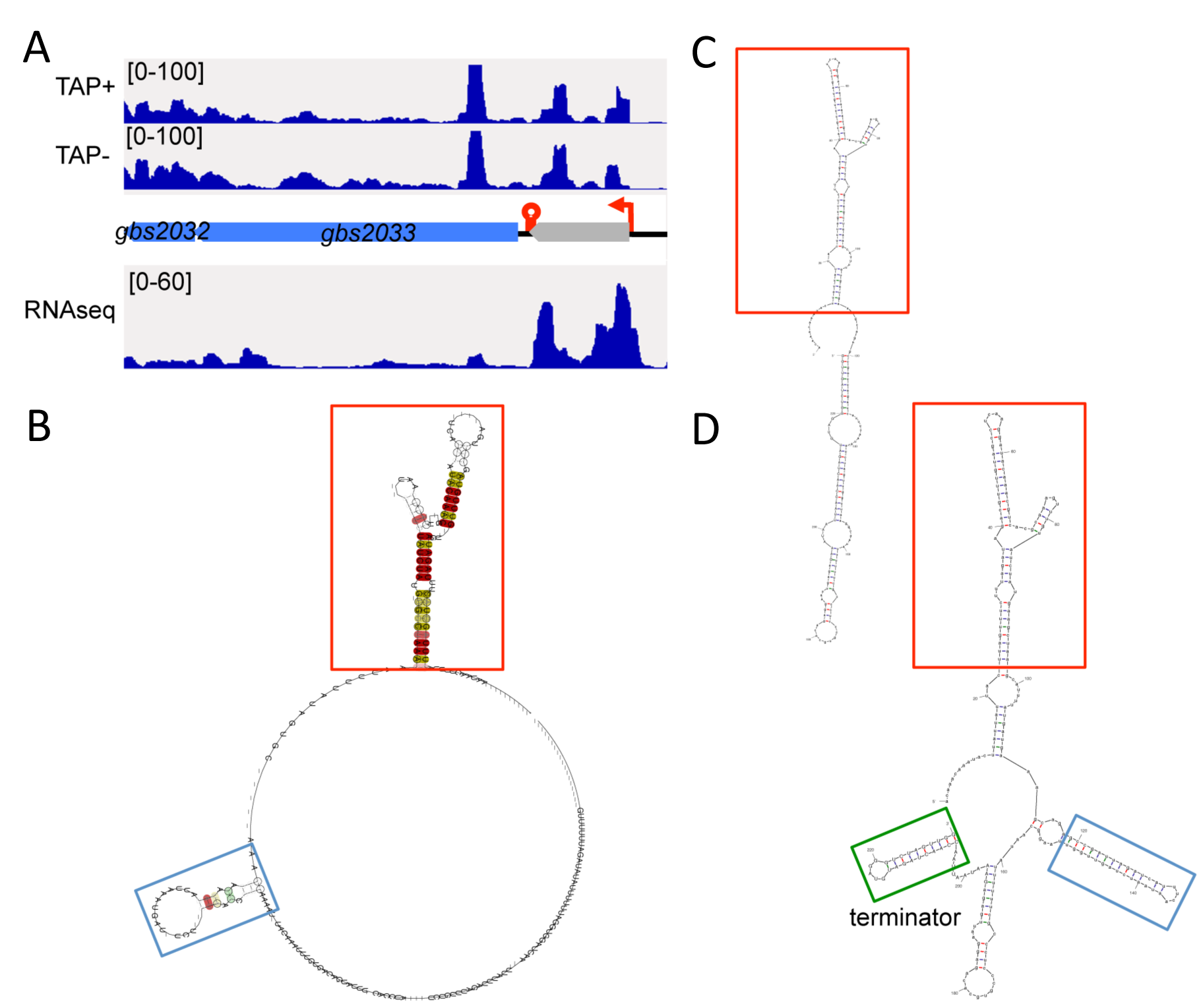

Supplement: Additional file 11: — Identification of a novel riboswitch upstream gbs2033. A. Identification from dRNA-seq and RNA-seq experiments. Reads aligning upstream gbs2033 were visualized by the IGV browser. The sequence of the sRNA resulting from transcription premature arrest at a rho-independent terminator is indicated as a grey arrow. B. Structure prediction by RNalifold, based on the alignment of 9 sequences similar to gbs2033 5′UTR in Lactobacillales and upstream the aroF gene in E. faecium (Additional file 10). The two folded and conserved structures predicted are indicated in red and blue boxes. C and D. Two alternative structures of gbs2033 putative riboswitch as determined by mfold. The two regions found by Rnalifold are indicated in red and blue boxes. The green box corresponds to a rho-independent terminator. [file 12864_2015_1583_MOESM11_ESM.tiff]

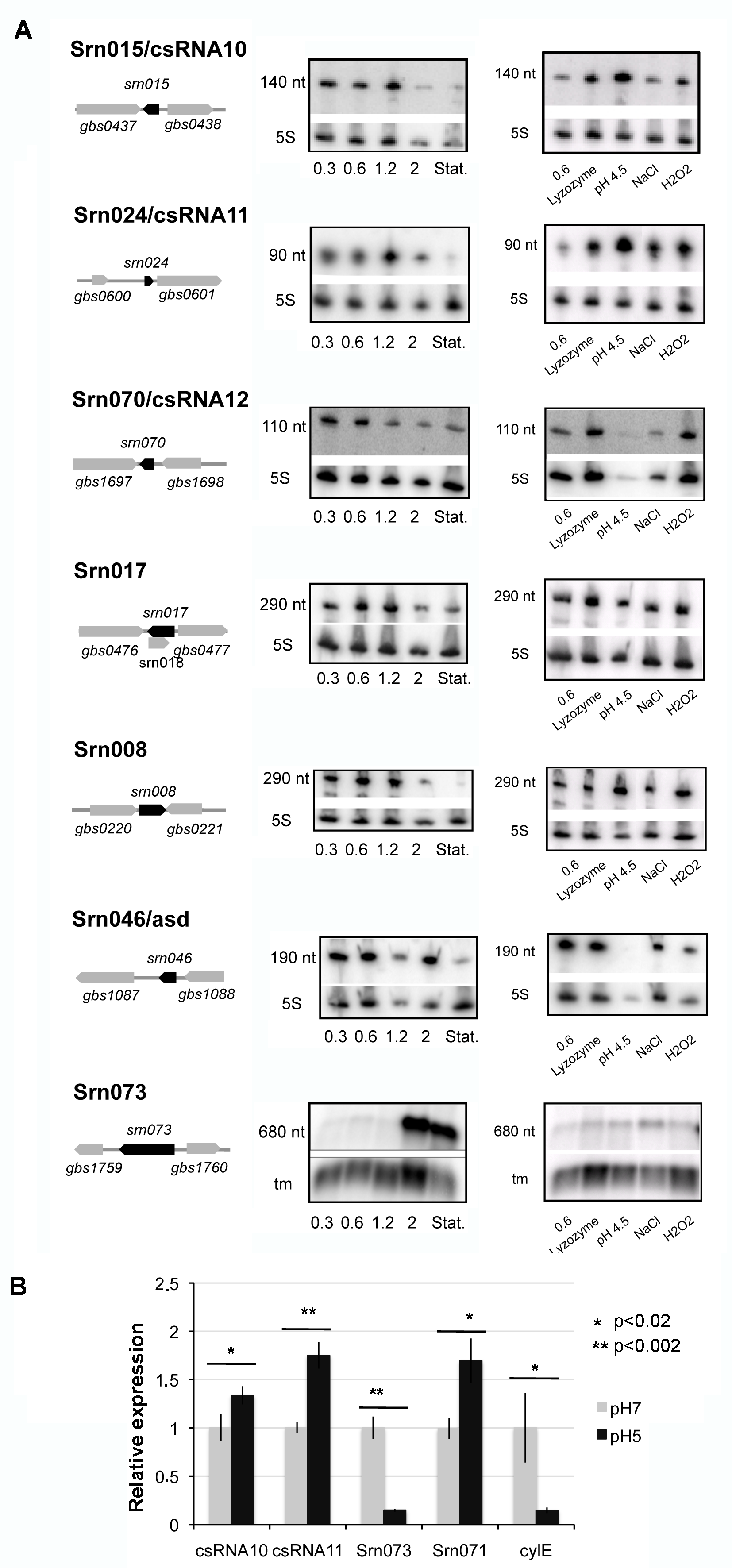

Supplement: Additional file 13: — Growth phase and stress-dependent expression of NEM316 sRNAs. A. Northern blot experiments showing the expression of seven ncRNAs (Snr015/csRNA10, Srn024/csRNA11, Srn070/csRNA12, Srn008, Srn017, Srn046/asd and Srn073) according to growth phase conditions or in response to various stresses. Total RNAs were prepared from cultures harvested at OD600: 0.3, 0.6, 1.2, 2 and late stationary phase (STAT) or grown to O.D600 = 0.6 and subjected to 15 min lysozyme (200 ng/ml), 30 min acid (pH 4.5), 20 min salt (NaCl 1 M) or 15 min oxidative (H202 4 mM) stresses. 5S RNA and tmRNA were used as loading controls. B. qRT-PCR experiments on four sRNAs (csRNA10, csRNA11, Srn073, Srn071) and the cylE gene. mRNAs were extracted from triplicate cultures of S. agalactiae bacteria grown in TH medium. Bacteria were harvested at O.D600 = 0.4 and resuspended in fresh TH adjusted or not to pH 5.0. Real-time PCR was performed on cDNA preparations using the SYBR green detection system (Applied Biosystems, Warrington, UK). Primers are listed in Additional file 17. Mean ± SD (N = 3). A p-value < 0.05 was considered as significant (unpaired bilateral Student’s t test). [file 12864_2015_1583_MOESM13_ESM.tif]

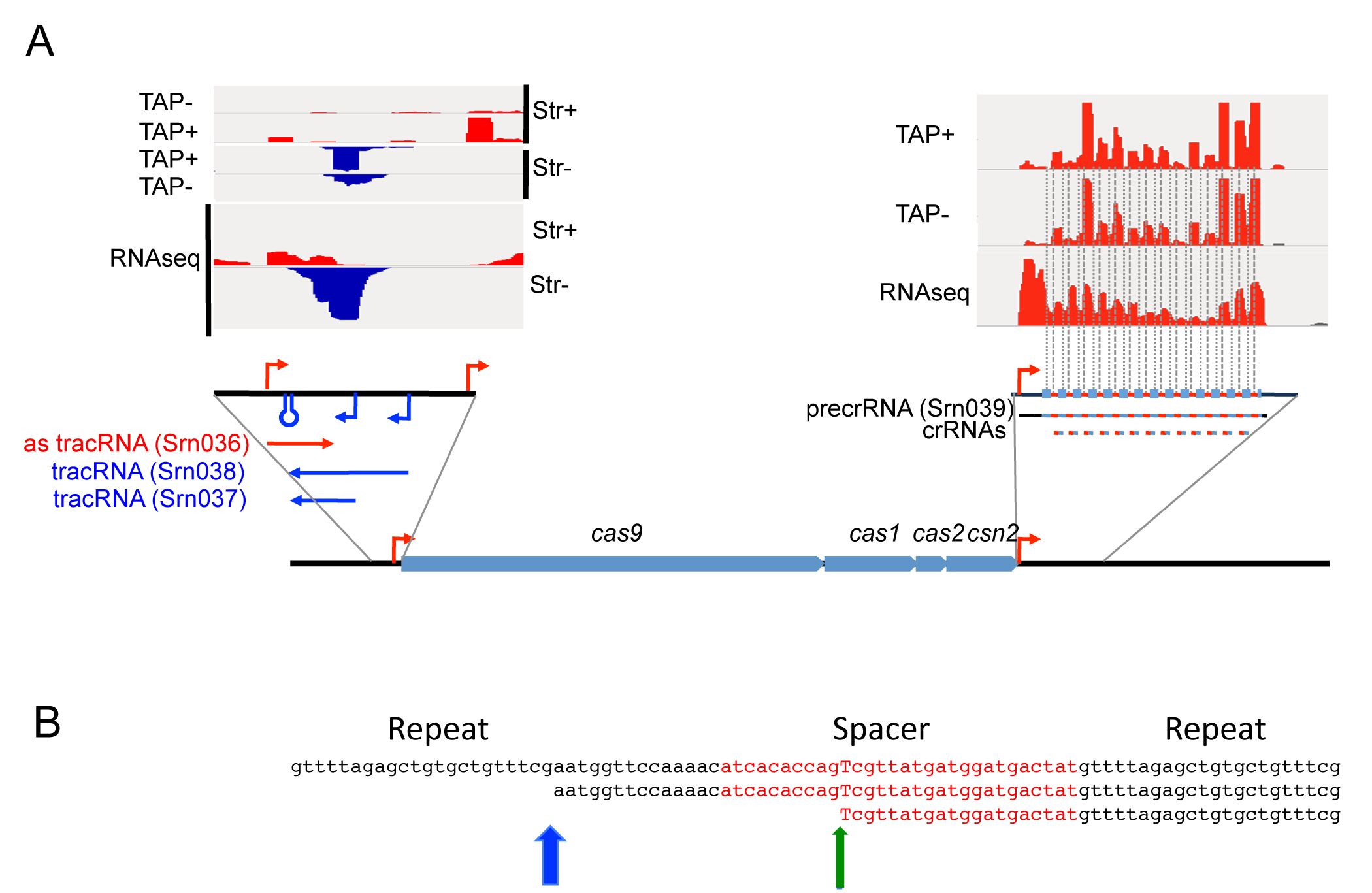

Supplement: Additional file 15: — Transcriptional organization of the CRISPR locus in S. agalactiae strain NEM316. A. Transcription and maturation profiles of the crRNA and tracrRNA. Combined results from the RNA-seq and dRNA-seq experiments allowed to define the TSS for the cas operon, for the precrRNA and for the two tracrRNAs as well as to characterize the maturation profile of the CRISPR array. In addition, they revealed a novel sRNA (Srn036) indicated by a red arrow, partially overlapping tracrRNA, indicated by blue arrows, in antisense orientation that might interfere with tracrRNA functions or regulation. B. Position of the maturation sites in crRNA. The large blue arrow indicates the maturation site resulting from hybridization with the tracrRNA and RNaseIII digestion, whereas the thin green arrow indicates the position of the second processing site of unknown origin. [file 12864_2015_1583_MOESM15_ESM.tiff]
