# Supplementary material for: Single nucleotide resolution RNA-seq uncovers new regulatory mechanisms in the opportunistic pathogen Streptococcus agalactiae
Source: BMC Genomics. 2015 May 30;16(1):419. doi: 10.1186/s12864-015-1583-4 (PMC4448216; doi:10.1186/s12864-015-1583-4)
Supplement: Additional file 9: — Alignment of DNA sequences similar to gbs1262 5′UTR. The DNA sequences in 14 Lactobacillales and upstream a potential tryptophan-related gene in F. nucleatum were extracted from Genbank. Accession numbers are given in the lower panel. Alignment was performed by using clustalW and a secondary structure was calculated with RNAalifold. In the alignment, sequence covariations supporting the consensus structure are marked by color: red marks pairs with no sequence variation; ochre and green mark pairs with 2 or 3 types of pairs, respectively. [file 12864_2015_1583_MOESM9_ESM.pdf]

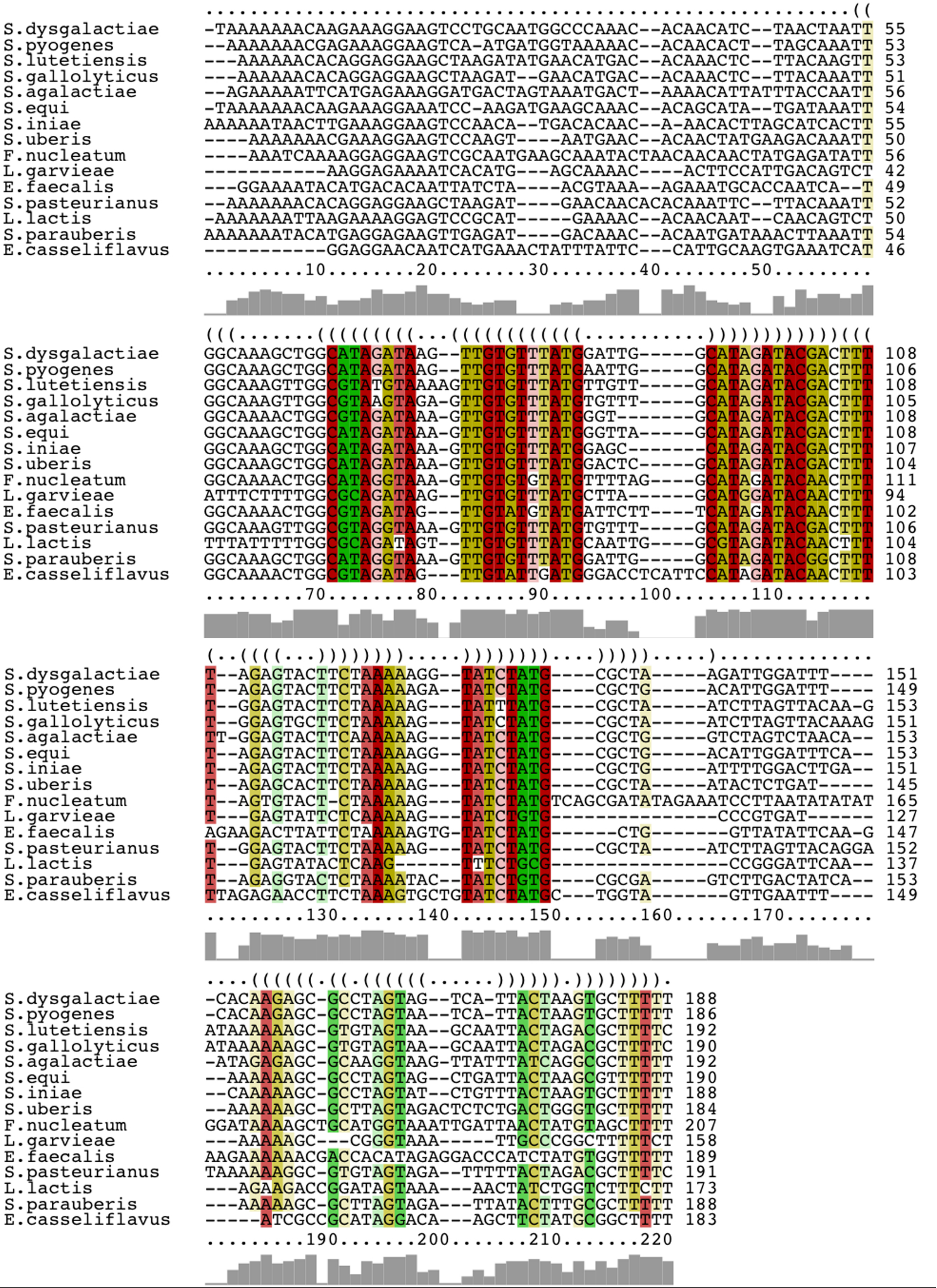

Abbreviation	species	Acc. number	downstream protein	Protein annotation
S.agalactiae	*Streptococcus agalactiae* NEM316	NC_004368.1	Gbs1262	putative ABC transport system substrate-binding protein
S.equi	*Streptococcus equi* subsp. equi 4047	emb|FM204883.1|	SEQ_1412	putative exported protein
S.iniae	*Streptococcus iniae* strain ISNO	gb|CP007587.1|	DW64_03725	peptide ABC transporter substrate-binding protein
S.uberis	*Streptococcus uberis* 0140J	emb|AM946015.1|	SUB1110	putative exported protein
S.gallolyticus	*Streptococcus gallolyticus* subsp. gallolyticus ATCC 43143	dbj|AP012053.1|	SGGB_0836	putative ABC transport system substrate-binding
S.pyogenes	*Streptococcus pyogenes* M1 GAS	gb|AE004092.2|	M5005_Spy_0743	ABC transporter substrate-binding protein
F.nucleatum	*Fusobacterium nucleatum* subsp. animalis 7_1	gb|CP007062.1|	FSDG_01183	tryptophan synthase beta chain
L.garvieae	*Lactococcus garvieae* ATCC 49156	dbj|AP009332.1|	LCGT_0748	amino acid transporter protein
E.faecalis	*Enterococcus faecalis* str. Symbioflor	emb|HF558530.1|	YP_007152865.1	ABC transporter, substrate binding protein
L. lactis	*Lactococcus lactis* subsp. cremoris KW2	gb|CP004884.1|	kw2_1881	amino acid permease
S.lutetiensis	*Streptococcus lutetiensis* 033	gb|CP003025.1|	KE3_0731	putative ABC transport system substrate-binding protein
S.pasteurianus	*Streptococcus pasteurianus* ATCC 43144	dbj|AP012054.1|	SGPB_0721	putative ABC transport system substrate-binding protein
S.parauberis	*Streptococcus parauberis* KCTC 11537	NC_015558	STP_0962	putative ABC transport system
S.dysgalactiae	*Streptococcus dysgalactiae* subsp. equisimilis RE378	NC_018712	GGS_1139	ABC transporter, substrate binding protein
E.casseliflavus	*Enterococcus casseliflavus* EC20	NC_020995	ECBG_00979	ABC transporter, substrate binding protein
