# Supplementary material for: Single nucleotide resolution RNA-seq uncovers new regulatory mechanisms in the opportunistic pathogen Streptococcus agalactiae
Source: BMC Genomics. 2015 May 30;16(1):419. doi: 10.1186/s12864-015-1583-4 (PMC4448216; doi:10.1186/s12864-015-1583-4)
Supplement: Additional file 10: — Alignment of DNA sequences similar to gbs2033 5′UTR. The DNA sequences in ten Lactobacillales and upstream the aroF gene in E. faecium were extracted from Genbank. Accession numbers are given in the lower panel. Alignment was performed by using clustalW and a secondary structure was calculated with RNAalifold. In the alignment, sequence covariations supporting the consensus structure are marked by color: red marks pairs with no sequence variation; ochre and green mark pairs with 2 or 3 types of pairs, respectively. [file 12864_2015_1583_MOESM10_ESM.pdf]

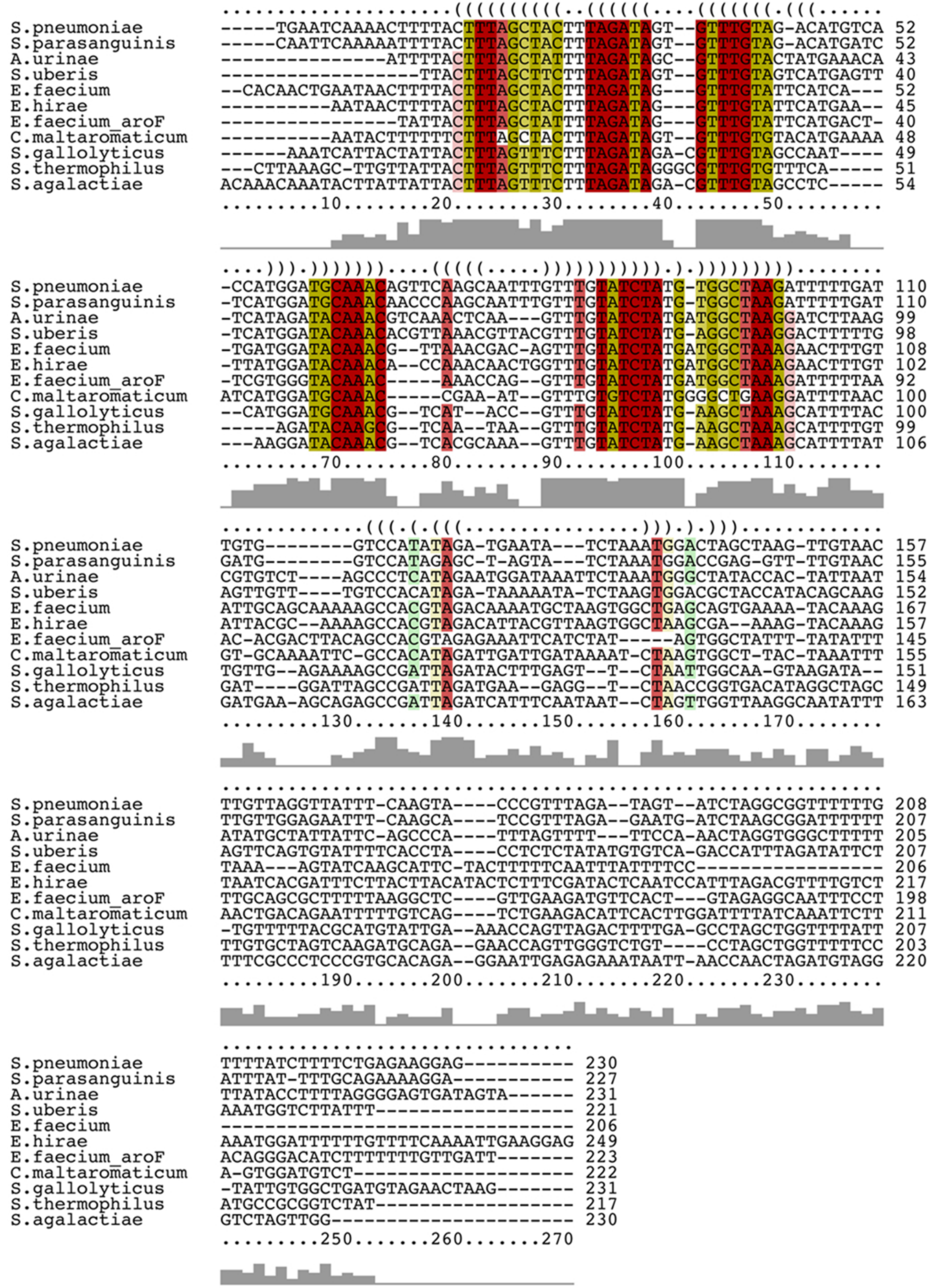

Abbreviation	species	Acc. number	downstream protein	Protein annotation
S.agalactiae	*Streptococcus agalactiae* NEM316	NC_004368.1	gbs2033	ABC transporter substrate-binding protein
S.pneumoniae	*Streptococcus pneumoniae* SPNA45	NC_018594.1	SPNA45_00365	substrate-binding protein (pseudogene)
S.gallolyticus	*Streptococcus gallolyticus* UCN34	NC_013798.1	GALLO_0122	ABC transporter substrate-binding protein
S.thermophilus	*Streptococcus thermophilus* LMG 18311	NC_006448.1	stu0201	ABC transporter substrate binding
S.uberis	*Streptococcus uberis* 0140J	emb|AM946015.1|	SUB0137	ABC transporter substrate-binding protein
C.maltaromaticum	*Carnobacterium maltaromaticum* LMA28	emb|HE999757.2|	BN424_2221	ABC transporter substrate-binding protein
E.hirae	*Enterococcus hirae* ATCC 9790	gb|CP003504.1|	EHR_05180	ABC-type uncharacterized transport system
A.urinae	*Aerococcus urinae* ACS120VCol10a	gb|CP002512.1|	HMPREF9243_1135	ABC transporter substrate binding protein
S.parasanguinis	*Streptococcus parasanguinis* FW213	NC_017905.1	Spaf_1564	ABC transporter substrate-binding protein
E.faecium	*Enterococcus faecium NRRL B-2354*	gb|CP003583.1|	M7W_2682	ABC superfamily ATP binding cassette
E.faecium_aroF	*Enterococcus faecium NRRL B-2354*	gb|CP004063.1|	M7W_1273	aroF:2-keto-3-deoxy-D-arabino-heptulosonate-7-phosphate synthase
