# Supplementary material for: Single nucleotide resolution RNA-seq uncovers new regulatory mechanisms in the opportunistic pathogen Streptococcus agalactiae
Source: BMC Genomics. 2015 May 30;16(1):419. doi: 10.1186/s12864-015-1583-4 (PMC4448216; doi:10.1186/s12864-015-1583-4)
Supplement: Additional file 16: — Bacterial strains used in the study. [file 12864_2015_1583_MOESM16_ESM.pdf]

Additional File 16: Bacterial strains

Strain	Description	Source or reference
NEM316	Serotype III isolated from neonate blood culture, ST23	[1]
Δ*covRS*	NEM2089 strain: NEM316 covSRΩaphA-3'; ΔcovSR, Km^R	[2]
Δ*RelRS*	NEM316 Δ*gbs1397-1398*	This study
Δ*ciaRH*	NEM316 Δ*ciaRH*	This study

References:
[1] Gaillot O, Poyart C, Berche P, Trieu-Cuot P (1997) Molecular characterization and expression analysis of the superoxide dismutase gene from *Streptococcus agalactiae*. Gene 204: 213-218.
[2] Lamy MC, Zouine M, Fert J, Vergassola M, Couve E, et al. (2004) CovS/CovR of group B streptococcus: a two-component global regulatory system involved in virulence. Mol Microbiol 54: 1250-1268.
