# Supplementary material for: Single nucleotide resolution RNA-seq uncovers new regulatory mechanisms in the opportunistic pathogen Streptococcus agalactiae
Source: BMC Genomics. 2015 May 30;16(1):419. doi: 10.1186/s12864-015-1583-4 (PMC4448216; doi:10.1186/s12864-015-1583-4)
Supplement: Additional file 17: — Oligonucleotides used in this study. [file 12864_2015_1583_MOESM17_ESM.pdf]

Additional file 17: Oligonucleotides used in this study

Oligonucleotide	Sequence (5' to 3')
**Mutant construction**	
*ciaR*_Eco	CTAGAATTCCGATGGGAATTTCAAACGAC
*ciaR*_Int1	GGCACGCCCGGGTGCTGCCGCGGAAAGACTCAAGTCATCTTC
*ciaH*_Int2	GCGGCAGCACCCGGGCGTGCCGATAATAAACCTAGAGGCAGT
*ciaH*_Bam	CAGGATCCGGCGCTCTGTTAAACTTTCG
*relR*_Eco	CGTCGGAATTCGTTACCTGTAACAGTAGATTCTGC
*relR*_int1	GGCACGCCCGGGTGCTGCCGCGACACGTGACATCTGTTCTTCATC
*relS*_int2	GCGGCAGCACCCGGGCGTGCCCATGATACCATTACCTTTGTCATT
*relS*_Bam	TCTAAGGATCCTGCCTCCAAATCTTCATC
**Probes for Northern blot**	
Srn008	ACTCAGGAGGCAACACCTCTACCTAATACCTTCAATAAGTATTTTT
Srn015	TCAGGAGATTTATGAAAAAGGAAAATAATTTAGGATAATATTTTCCTA
Srn017	TATAAACATGAGTGTTAAGACCTCAAAGGTAGACAAATCTAGCTCCTC
Srn024	ATATTAGGAGATTGTTCTTCATCAGTAAAACAAAGAACATGAAAA
Srn046	GAAATTACAAGTAGTAGTAAGCTAAGGGCACCTTTTCAAGTGTGACAA
Srn070	AACTTTCTGGTTTTTCGTAGTTGCAGTATGCTTTCAAAATAGAAAAAAC
Srn073	TCTGTCTTCCTTAAATTATATAAGGACTTATGTGTCACCGTTACT
**Primers for qRT-PCR**	
qcrRNA10_F	GCGGTTTATCCTAGCCGTTA
qcrRNA10_R	GGGTTGGAAAAAGGGTTGAG
qcrRNA11_F	CTTTTCTTTTCATGTTCTTTGTTTT
qcrRNA11_R	TGAGTTGCAAATATTAGGAGATTG
qcrSrn073_F	TGTTGCAAACAGTCACAAGGA
qcrSrn073_R	AGCATGCGCTAATGGTAATGA
qcrSrn071_F	ATCACAGATGTGGAGGTATGTTATG
qcrSrn071_R	AGTCGGTAAGAAAAATCTATTCTGAAA
cylE_F	CCAGACGGTAGGCCTTTAACT
cylE_R	AGTGATTGCCTGTCCACTACG
